# Quantum Tunneling Facilitates Water Motion across the Surface of Phenanthrene

**DOI:** 10.1021/jacs.3c04281

**Published:** 2023-07-26

**Authors:** Donatella Loru, Amanda L. Steber, Cristóbal Pérez, Daniel A. Obenchain, Berhane Temelso, Juan C. López, Melanie Schnell

**Affiliations:** † Deutsches Elektronen-Synchrotron DESY, Notkestr. 85, 22607 Hamburg, Germany; ‡ Division of Information Technology, College of Charleston, Charleston, South Carolina 29424, United States; § Departamento de Química Física y Química Inorgánica, Facultad de Ciencias, Universidad de Valladolid, 47011 Valladolid, Spain; ∥ Institut für Physikalische Chemie, Christian-Albrechts-Universität zu Kiel, Max-Eyth-Straße 1, D-24118 Kiel, Germany

## Abstract

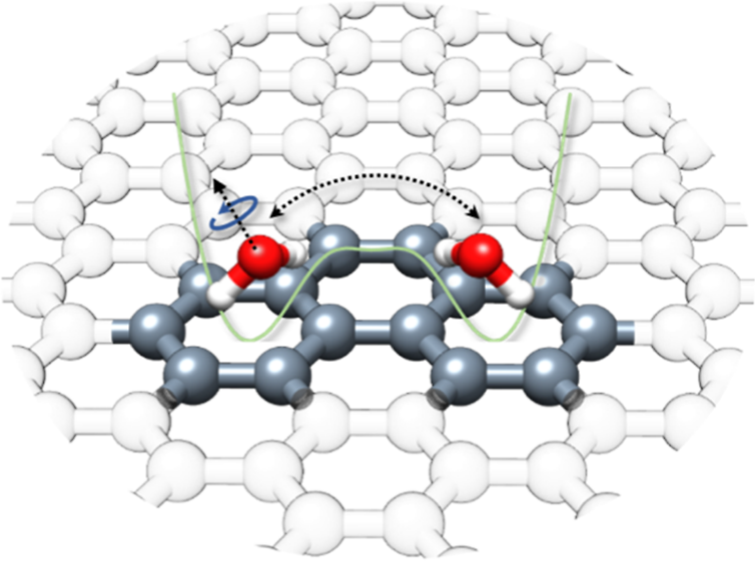

Quantum tunneling is a fundamental phenomenon that plays a pivotal role in the motion and interaction of atoms and molecules. In particular, its influence in the interaction between water molecules and carbon surfaces can have significant implications for a multitude of fields ranging from atmospheric chemistry to separation technologies. Here, we unveil at the molecular level the complex motion dynamics of a single water molecule on the planar surface of the polycyclic aromatic hydrocarbon phenanthrene, which was used as a small-scale carbon surface-like model. In this system, the water molecule interacts with the substrate through weak O–H···π hydrogen bonds, in which phenanthrene acts as the hydrogen-bond acceptor via the high electron density of its aromatic cloud. The rotational spectrum, which was recorded using chirped-pulse Fourier transform microwave spectroscopy, exhibits characteristic line splittings as dynamical features. The nature of the internal dynamics was elucidated in great detail with the investigation of the isotope-substitution effect on the line splittings in the rotational spectra of the H_2_^18^O, D_2_O, and HDO isotopologues of the phenanthrene–H_2_O complex. The spectral analysis revealed a complex internal dynamic showing a concerted tunneling motion of water involving its internal rotation and its translation between the two equivalent peripheral rings of phenanthrene. This high-resolution spectroscopy study presents the observation of a tunneling motion exhibited by the water monomer when interacting with a planar carbon surface with an unprecedented level of detail. This can serve as a small-scale analogue for water motions on large aromatic surfaces, i.e., large polycyclic aromatic hydrocarbons and graphene.

## Introduction

Water is a simple molecule crucial in biological and chemical systems. With the development of more sophisticated experimental and theoretical methods, our understanding of water’s role in biological and chemical systems has changed. We no longer think of water as solely a spectator and solvent but as directly influencing numerous phenomena—a true paradigm change.

Due to its versatile nature in which it has the ability to act both as a hydrogen-bond donor and acceptor, water can interact with a multitude of substrates by forming a variety of hydrogen bonds. Each individual water molecule can form up to four intermolecular interactions, resulting in highly flexible networks, which adapt to the structure of the substrate and give rise to a variety of water-binding motifs. While in the liquid phase, ultrafast rearrangements of the hydrogen-bond network appear on femtosecond to picosecond time scales,^1,2^ in the gas phase, rich internal dynamics play an important role in isolated water clusters, such as in the observed water hexamer^3^ as well as in various solute–water clusters.^4−7^

Currently, there is a significant focus on the interaction and dynamics of water on carbon surfaces, e.g., graphene, which have a critical role in various chemical and physical phenomena in our everyday lives and in scientific and technological processes,^8^ including but not limited to ice nucleation,^9^ electrochemistry,^10^ corrosion,^11^ catalysis,^12^ separation technology,^13,14^ atmospheric chemistry,^15^ and interstellar dust grains.^16^ An accurate understanding on a molecular level of water interaction and dynamics at the carbon surface is fundamental to comprehend these phenomena. However, a complete picture is yet to be developed. The study of the motion of a single water molecule on a carbon surface is a challenging task experimentally and has led to a lack of benchmarking experimental data. Two main reasons for this are the strong tendency among water molecules to establish hydrogen bonds, resulting in rapid formation of water clusters, and the high mobility of water protons, which makes it particularly challenging for imaging techniques to determine the position of the hydrogen atoms as well as the overall orientation of the water molecule. It was only recently that our understanding of the diffusion of water molecules on carbon surfaces, e.g., graphene, started to unfold, with the experimental investigation of the motion of a single water molecule on graphene using helium spin-echo techniques.^9^ This experiment showed that the water motion on graphene takes place through a hopping motion of the water molecule between the centers of the graphene hexagons.

Here, using high-resolution broadband rotational spectroscopy, we investigated with unprecedented details the dynamics of a single water molecule on the aromatic surface of phenanthrene (Phe), a planar three-ring polycyclic aromatic hydrocarbon (PAH) that can be considered as a small-scale carbon interface-like model of pristine graphene, which is planar and consists of only carbon atoms. Our study revealed that the water molecule moves across the carbon surface via a concerted tunneling motion involving the internal rotation of the water molecule and its translation motion between two equivalent aromatic rings. In high-resolution gas-phase rotational spectroscopy, internal dynamics of a molecular system are generally presented as characteristic spectral features. These features can be as simple as a rotational transition splitting into two components, or they can be more complicated spectral patterns. The case of the former can be observed, e.g., when a water molecule undergoes an internal rotation around its *C*_2_ symmetry axis,^17−19^ and the resulting doublets for each rotational transition have a recognizable 3:1 intensity ratio due to nuclear spin statistics. An example of the latter case is the doublets-of-triplets splitting pattern observed in the rotational spectroscopy study of the water hexamer,^3^ which was attributed to a tunneling motion involving the simultaneous breaking of two hydrogen bonds in the water cluster.

In a previous comparative study between Phe and its nitrogen-substituted analogue phenanthridine (Pan), we focused on the intermolecular interactions and structure of a series of water clusters, up to PAH–(H_2_O)_3_.^20^ In contrast to the molecular clusters Phe–(H_2_O)_2_ and Phe–(H_2_O)_3_ as well as Pan–(H_2_O)_*n*_, *n* = 1–3, the rotational spectrum of the monohydrated cluster of phenanthrene shows characteristic line splittings due to rich internal dynamics, which were neither analyzed nor discussed in ref (20). Here, we present a detailed analysis and interpretation of the mechanism underlying these internal dynamics, based on additional spectroscopic measurements, in-depth computations, and modeling. We report new spectroscopic results of the water isotopologues D_2_O and HDO, as well as a complete theoretical treatment to disentangle the complex nature of the motion of water on the Phe surface. The observed differences in the experimental splitting patterns for the water isotopologues provide valuable information to rationalize and model the tunneling pathways. The spectroscopic analysis is supported by quantum-chemical calculations and Meyer’s flexible model,^21^ which was used to assess the tunneling pathways.

## Results and Discussion

**Figure 1:**
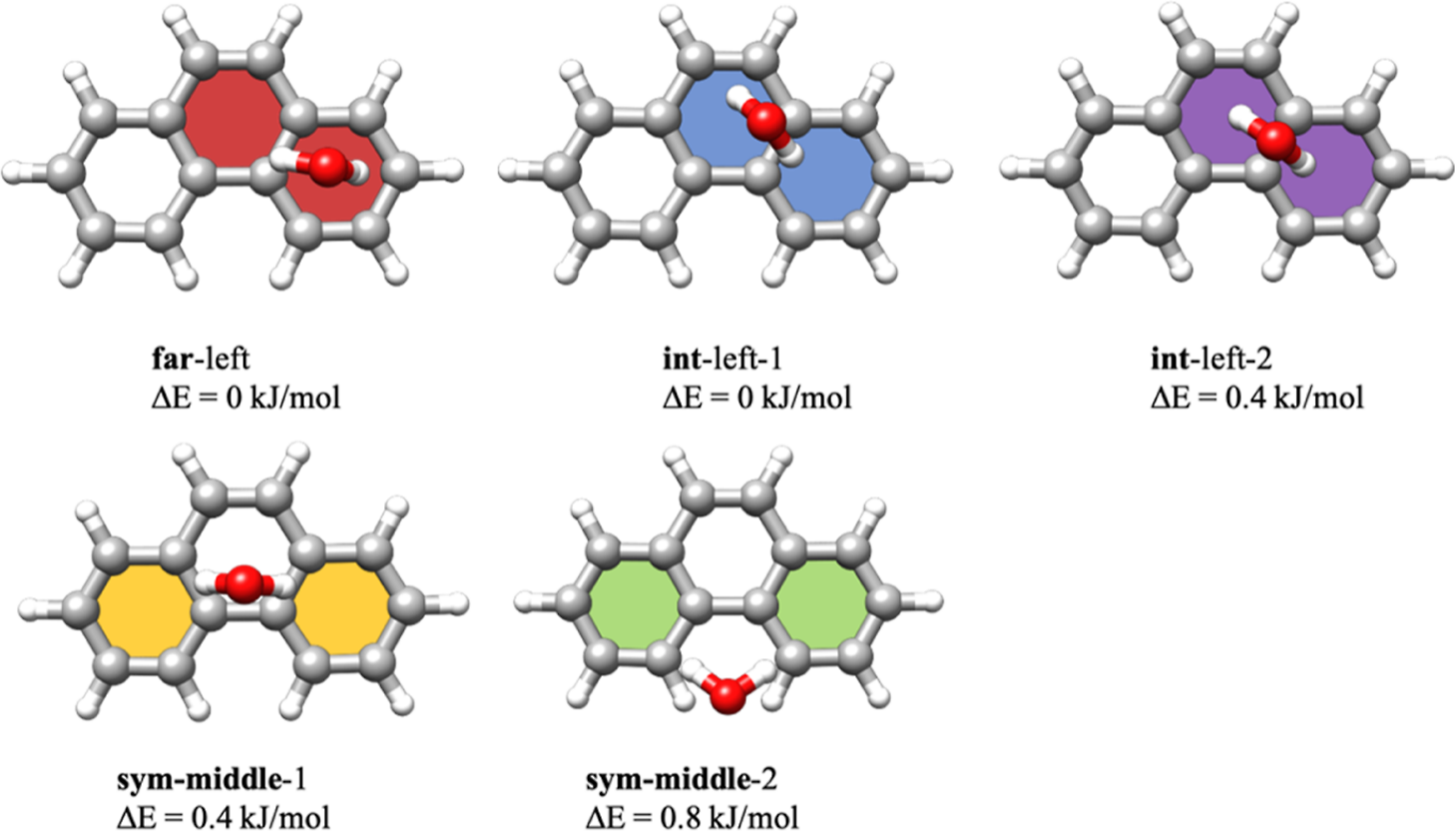
Optimized structures calculated at the PBEh-3c level of theory and relative single-point energies calculated at the DLPNO-CCSD(T) level of theory of the five unique isomers of the Phe–H_2_O cluster. For both **far-** and **int-**isomers, only the geometries in which the water molecule is located above the left aromatic ring of phenanthrene are showcased.

In the Phe–H_2_O complex, the water molecule interacts with the substrate via O–H···π interactions and can thus be expected to dock in a number of different locations. As a consequence, different geometries of its monohydrated complex, connected via only low interconversion barriers, are possible. However, the overall *C*_2*v*_ symmetry of phenanthrene results in equivalent interaction positions and thus reduces the number of unique isomers (Figure 1).

A computational study of the structures of the different complexes highlighting the preferred binding sites of water to phenanthrene was already reported in our previous publication, where we compared the binding sites between two similar yet compositionally distinct PAHs. This computational study pointed to an extremely shallow potential energy surface (PES) with the low-energy isomers connected by low barriers. Here, we reinvestigated the PES of the Phe–H_2_O cluster using another systematic approach involving Coalescence-Kick software.^22,23^ The new conformational search uncovered the presence of eight isomers: five unique and nearly isoenergetic isomers that differ in the position where the water molecule interacts with phenanthrene and three others related to them by symmetry. The five unique minima are presented in Figure 1. The isomers have been labeled depending on the position of water relative to the phenanthrene molecule as sym-middle-1, sym-middle-2, int-left-1, int-left-2, and far-left, with the symmetry-equivalent analogues of the far-left, int-left-1, and int-left-2 isomers being named as far-right, int-right-1, and int-right-2. The structures have been optimized using a density functional theory method based on a composite electronic structure approach (PBEh-3c)^24^ with correction to dispersion (D3BJ),^25,26^ geometrical counterpoise correction (gCP),^27^ and basis set incompleteness error (BSIE) implemented in the ORCA software.^28,29^ Frequency calculations have been performed at the same level of theory to verify that the isomers of the Phe–H_2_O cluster are real minima in the PES. To obtain more accurate energies, single-point energy calculations at the DLPNO-CCSD(T)^30^ level of theory using a complete basis set (CBS) extrapolation based on the calculations with the cc-pVDZ and cc-pVTZ basis sets^31^ [denoted as CBS(2/3)], and done in the automatic procedure, have been performed on the optimized geometries. Quantum-chemical spectroscopic parameters are summarized for the five unique isomers in Table S1. The theoretical rotational constants are similar for all the isomers. For all of them, with the exception of the sim-middle-2 isomer, the predominant component of the dipole moment resides along the *c* principal axis, which arises from the water molecule located above the phenanthrene surface. In the sym-middle-1 isomer, the *C*_2_ symmetry axis of the water molecule almost coincides with the *c*-inertial axis of phenanthrene, thus explaining the predominance of the μ_*c*_ dipole moment component and only negligible values for μ_*a*_ and μ_*b*_. In the sym-middle-2, int-left, and far-left isomers, the *C*_2_ symmetry axis of the water molecule is tilted with respect to the *c* inertial axis of phenanthrene (Figure 1), yielding a non-negligible μ_*b*_ dipole moment component for the rigid equilibrium structures of the sym-middle-2 and int-left/right isomers and a non-negligible μ_*a*_ dipole moment component for the rigid equilibrium structure of the far-left/right isomer. These differences in dipole-moment components can support the assignment of the spectroscopically observed species.

The experimental rotational spectrum of the Phe–H_2_O complex was recorded in the 2–8 GHz frequency range (Figure S1) using the COMPACT spectrometer, which has been described in detail elsewhere.^32,33^ Individual measurements were performed for the different isotopologues Phe–H_2_^16^O, Phe–H_2_^18^O, Phe–D_2_O, and Phe–HDO, respectively. The experimental spectra of Phe–H_2_^16^O and Phe–H_2_^18^O were already presented in our earlier work.^20^ Detailed information about the experimental conditions can be found in the Supporting Information.

The experimental rotational spectrum of Phe–H_2_^16^O exhibits both ^*a*^*R*- and ^*c*^*R*-branch transitions as well as some ^*c*^*Q*-branch transitions. Due to the structural similarities between the five isomers, the assignment of the spectrum based on a comparison between experimental and theoretical rotational constants was nontrivial. Nevertheless, the observation of *a*-type transitions leads to the assignment of the experimental rotational spectrum of the far-isomer, being the only one for which a non-negligible *a*-component of the dipole moment is predicted by theory (Table S1).

**Figure 2:**
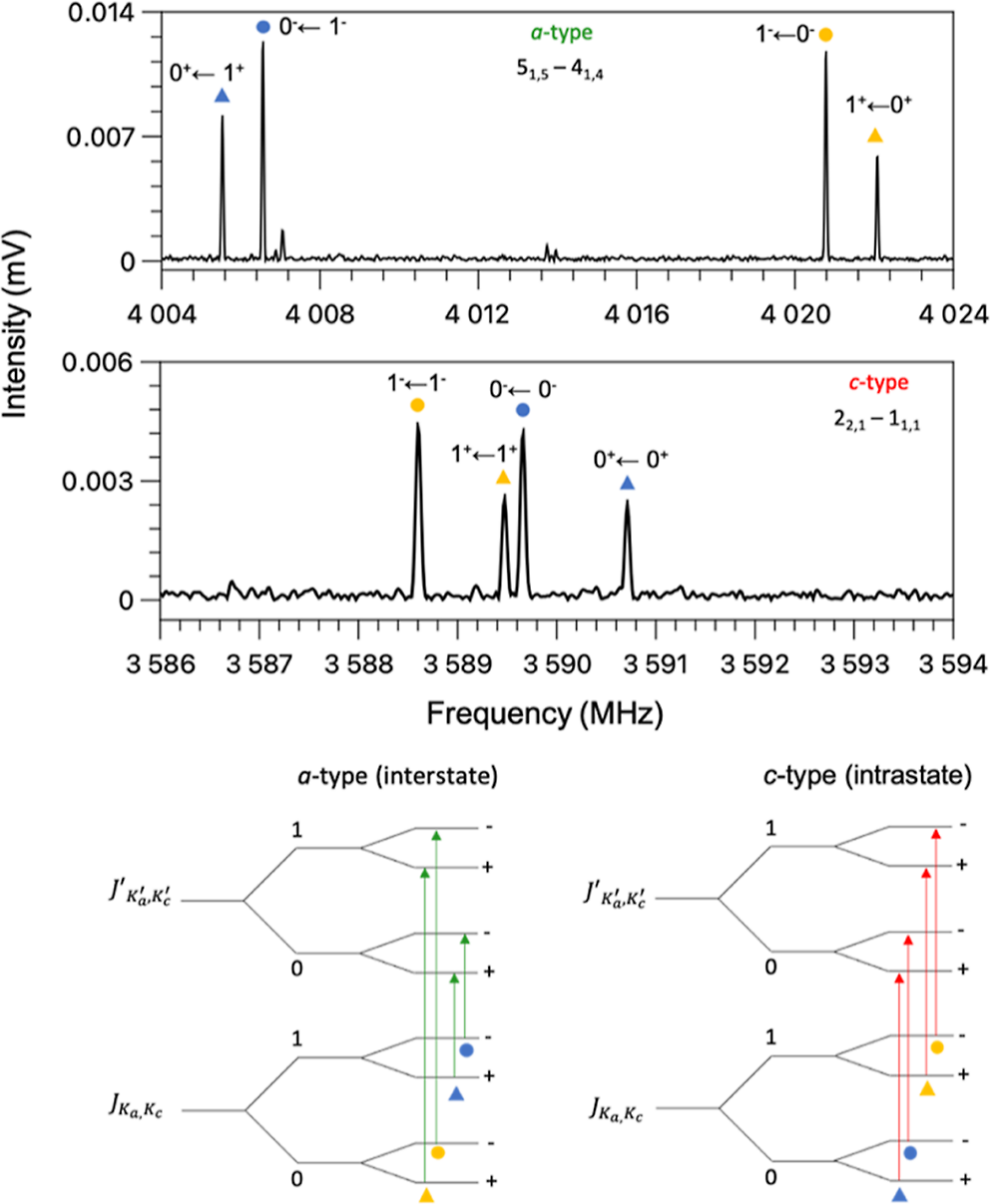
Experimental spectral pattern observed for transitions of *a*-type (top) and of *c*-type (bottom). Markers of the same color indicate the doublets arising from the *C*_2_ internal rotation of the water molecule. Markers of the same shape (circles or triangles) indicate the doublets arising from the tunneling motion of the water molecule between the two equivalent isomers of the Phe–H_2_O complex: far-left and far-right. The two transitions are labeled following the notation *J*_*Ka*,*Kc*_ ← *J*_*K*′*a*,*K*′*c*_^′^. For each component of the tunneling splitting, the lower and upper tunneling states are indicated as 0^+^, 0^–^, 1^+^, and 1^–^. A schematic of the interstate *a*- and intrastate *c*-type transitions is also shown at the bottom of the figure. The schematic of the transitions is not drawn to scale.

Both *a*- and *c*-type transitions exhibit a complex splitting pattern. They appear as a quartet (Figure 2) in which lines can be gathered into two doublets depending on their relative intensity ratios of ∼2.2:1 or ∼1:1, respectively. In monohydrated complexes, a splitting of the lines into doublets exhibiting an intensity ratio of 3:1 is often observed, and it is commonly generated by the internal rotation of water around its *C*_2_ internal symmetry axis in a two-fold periodic potential function. This motion exchanges two equivalent hydrogen atoms and splits the rotational transitions in two components with an intensity reflecting the Fermi nuclear spin statistical weights. A similar splitting has been previously observed in the microwave spectra of monohydrated clusters of solutes of different nature including PAHs, such as acenaphthene^17^ and corannulene^34^ to name a few, and it has been rationalized in terms of a low barrier for the *C*_2_ internal rotation of the water molecule. For the far-isomer of the phenanthrene–H_2_O complex, the barrier to this motion, which takes place through the rupture of weak O–H···π hydrogen bonds, has been calculated to be approximately 2 kJ/mol using the nudged elastic band (NEB) method^35^ implemented in Orca software.^28,29^ This value can be considered an upper value.

The 1:1 splitting arises from a second large amplitude motion, which involves a tunneling of the entire water molecule between the two equivalent geometries of the far-isomer (far-left and far-right). Presumably, the minimum energy pathway for this motion passes through the sym-middle conformation with *C*_*s*_ symmetry, which can be identified as the origin of the vibrational coordinate. For this isomer, the symmetry plane is coincident with the σ_*bc*_ inertial plane. Therefore, the μ_*a*_ component of the electric dipole moment behaves in a similar fashion to the vibrational coordinate. Both are antisymmetric with respect to the reflection at this plane. As a consequence, μ_*a*_ inverts its sign when the water molecule tunnels from the right to the left configuration of the far-isomer. This implies that the *a*-type transitions occur as interstate transitions and connect vibrational states of different symmetries crossing from the lower to the upper or the upper to the lower tunneling states (Figure 2). The corresponding doublet lines occur with almost constant separation corresponding to 2 times the value of the tunneling splitting and provide a direct measure of the vibrational energy difference between the tunneling states. The four tunneling states have been labeled using the notation 0^+^/0^–^ and 1^+^/1^–^, with 0 and 1 being the quantum numbers denoting the two tunneling states generated by the water translation, and + and – being applied to denote the water internal rotation.

For each pair of states, 0^+^/1^+^ or 0^–^/1^–^, the measured transition frequencies have been satisfactorily fitted by using the following two-state coupled Hamiltonian
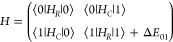
1*H*_*R*_ is based on the *S*-reduction of Watson’s Hamiltonian and represents the rotational and the centrifugal distortion Hamiltonians for the *v* = 0 and *v* = 1 tunneling states. *H*_*C*_ is the rotational operator of the Coriolis coupling terms connecting the vibrational states and corresponding to
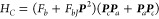
2Δ*E*_0^+^1^+^/0^–^1^–^_ is the difference in vibrational energy between the two tunneling states generated by water’s translational motion. The need of the *F*_*b*_ Coriolis term to fit the rotational transitions indicates that the large amplitude motion of the water molecule creates an angular momentum along the *b* inertial axis.

All the transitions were fitted using SPFIT/SPCAT implemented in Pickett’s program.^36^ With respect to our previous study, in which only an average of the rotational parameters was reported, we have reanalyzed the spectra to give correct assignments and to account properly for the internal motion coupling effects. In the present work, we present accurate spectroscopic constants for each of the four tunneling states as well as information on the vibrational energy difference between the tunneling states, Δ*E*. The obtained experimental spectroscopic parameters are reported in Table 1. Measured rotational transitions and the corresponding quantum numbers are reported in Table S9 of the Supporting Information.

**Table 1:**
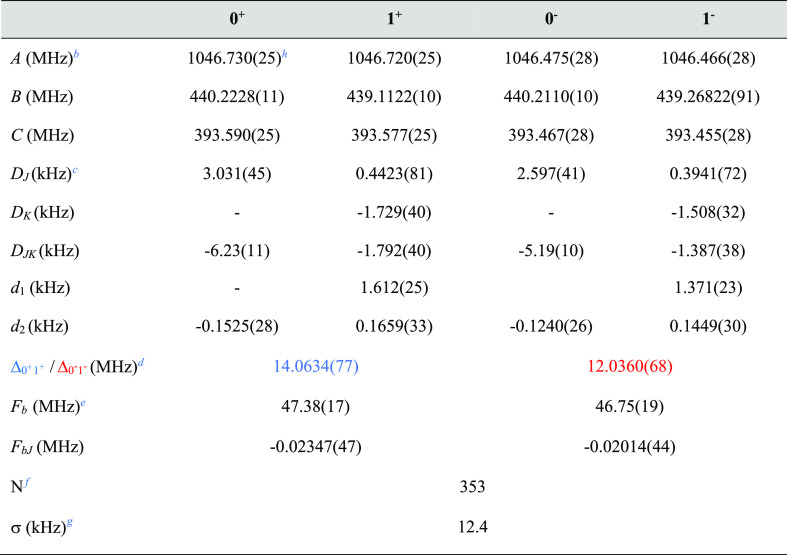
Experimental Spectroscopic Constants of the Far-Left/Right Isomer of the Phenanthrene–H_2_O Complex for the Four Different Tunneling States^a^

a The experimental rotational transitions were fit using the two-state coupled Hamiltonian following eq 1.

b *A*, *B*, and *C* are the rotational constants.

c *D*_*J*_, *D*_*K*_, *D*_*JK*_, *d*_1_, and *d*_2_ are the centrifugal distortion constants.

d Δ_0^+^1^+^_ and Δ_0^–^1^–^_ are the differences in vibrational energy between the two tunneling states 0^+^ and 1^+^ and 0^–^ and 1^–^, respectively.

e *F*_*b*_ and *F*_*bJ*_ are the Coriolis coupling terms.

f *N* is the number of lines in the fit.

g σ is the root-mean-square deviation of the fit.

h Standard errors within parentheses are expressed in units of the last two digits.

Isotopic substitution in different atoms is expected to affect the observed tunneling patterns in their respective spectra due to changes in mass and/or symmetry breaking. To confirm and further elucidate the proposed tunneling motions of the water molecule on the phenanthrene surface, we recorded the rotational spectra of the Phe–H_2_^18^O, Phe–D_2_O, and Phe–HDO isotopologues. Phe–H_2_^18^O and Phe–D_2_O complexes were formed by using isotopically enriched samples of H_2_^18^O and D_2_O water, respectively, whereas the Phe–HDO complex was formed by using a 1:1 mixture of H_2_O and D_2_O, which is known to result in fast proton exchange. For isotopologues for which the *C*_2*v*_ symmetry of water is kept, qualitatively similar tunneling motions are expected.

**Figure 3:**
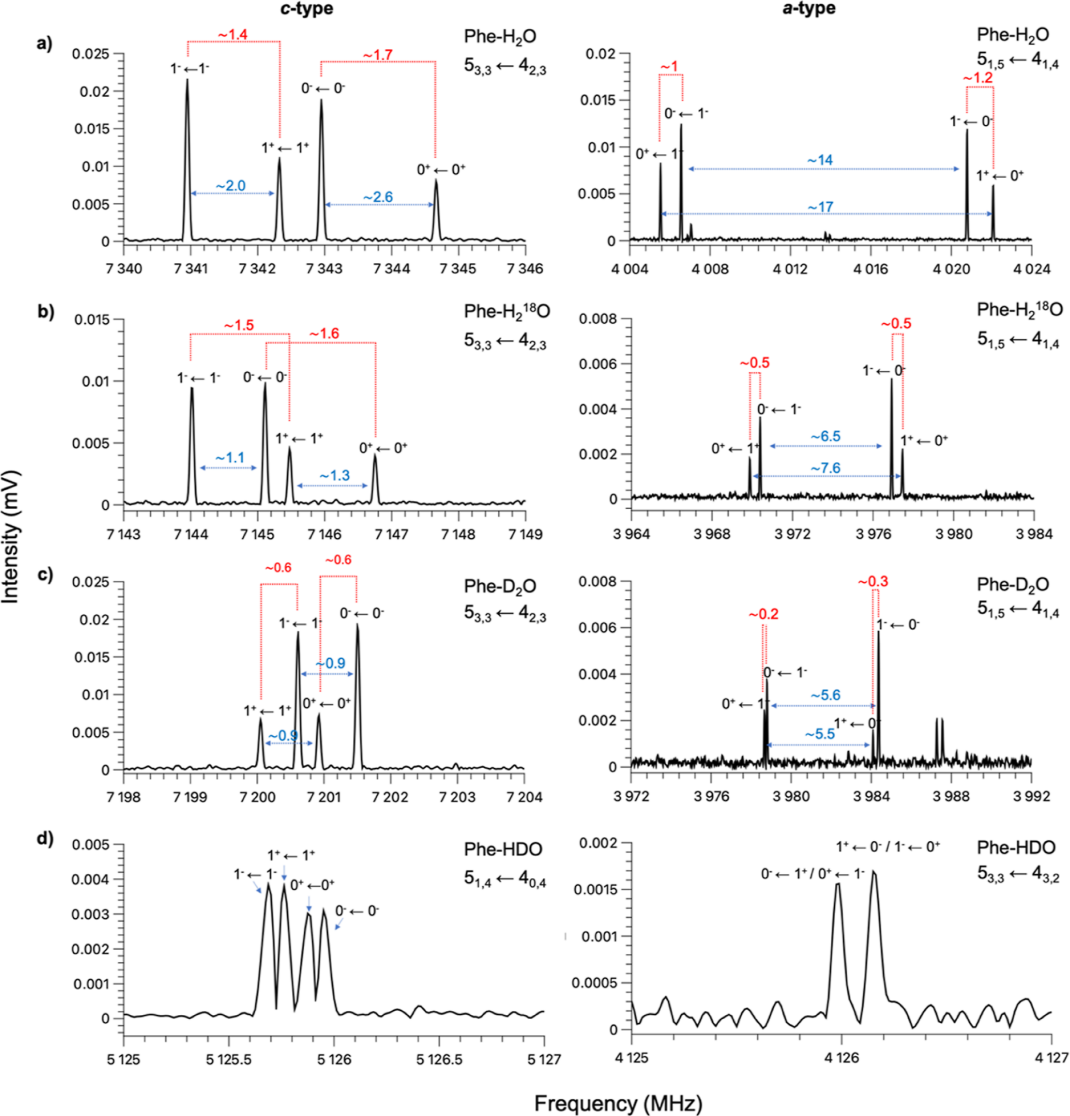
Left: sections of the experimental spectra showing the *c*-type transition 5_3,3_–4_2,3_ of Phe–H_2_O (a), Phe–H_2_^18^O (b), and Phe–D_2_O (c) and the *c*-type transition 5_1,4_–4_0,4_ of Phe–HDO (d). Right: sections of the experimental spectra showing the *a*-type transition 5_15_–4_14_ of Phe–H_2_O (a), Phe–H_2_^18^O (b), and Phe–D_2_O (c) and the *a*-type transition 5_3,3_–4_3,2_ of Phe–HDO (d). Red dashed lines connect the two tunneling states generated by *C*_2_ internal rotation of the water molecule, while the blue arrows connect the two tunneling states arising from water’s translation motion. The two transitions are labeled following the notation *J*_*Ka*,*Kc*_ ← *J*_*K*′*a*,*K*′*c*_^′^. For each component of the tunneling splitting, the lower and upper tunneling states are indicated as 0^+^, 0^–^, 1^+^, and 1^–^. The magnitude of the splittings is given in megahertz.

Representative *a*- and *c*-type transitions arising from the three isotopologues are showcased in Figure 3. The rotational spectra of phenanthrene–H_2_^18^O and phenanthrene–D_2_O exhibit the same characteristic fine structure, in terms of the number of components and intensity ratio, as the parent species, and they have been analyzed using the same two-state coupled Hamiltonian. The experimental spectroscopic parameters determined for all the investigated isotopologues are reported in the Supporting Information.

A closer inspection of the *c*-type transitions in the Phe–H_2_^18^O spectrum shows that the splitting between lines showing a 1:1 intensity ratio is reduced upon ^18^O isotopic substitution, whereas the splitting between the components showing a ∼2.2:1 intensity ratio remains almost unvaried. This confirms the motion assignment because the oxygen atom is significantly involved in the water translation, but it does not participate in the internal rotation of water around its *C*_2_ symmetry axis. Substitution of both hydrogen atoms with deuterium reduces the splitting between all four components, thus indicating that the hydrogen atoms are involved in both motions.

The *a*-type transitions show a reduced splitting between all four components, also in the case of Phe–H_2_^18^O (Figure 3). This is because the *a*-type transitions are interstate transitions and the splitting mainly depends on the vibrational energy difference between the tunneling states. This makes the effect of isotopic substitution on the splitting less evident than that for transitions of *c*-type. For the *c*-type transitions, the splitting is mainly influenced by the vibrational dependence of the rotational constants and by the Coriolis coupling.

Substitution of one of the water’s hydrogen atoms with deuterium breaks its *C*_2*v*_ symmetry and, therefore, is expected to quench both the translational motion and the *C*_2_ internal rotation of the water molecule. However, line splitting in both *a*- and *c*-type transitions is still observed in the rotational spectrum of the Phe–HDO complex (Figure 3), thus providing useful information to disentangle the respective tunneling pathways.

**Figure 4:**
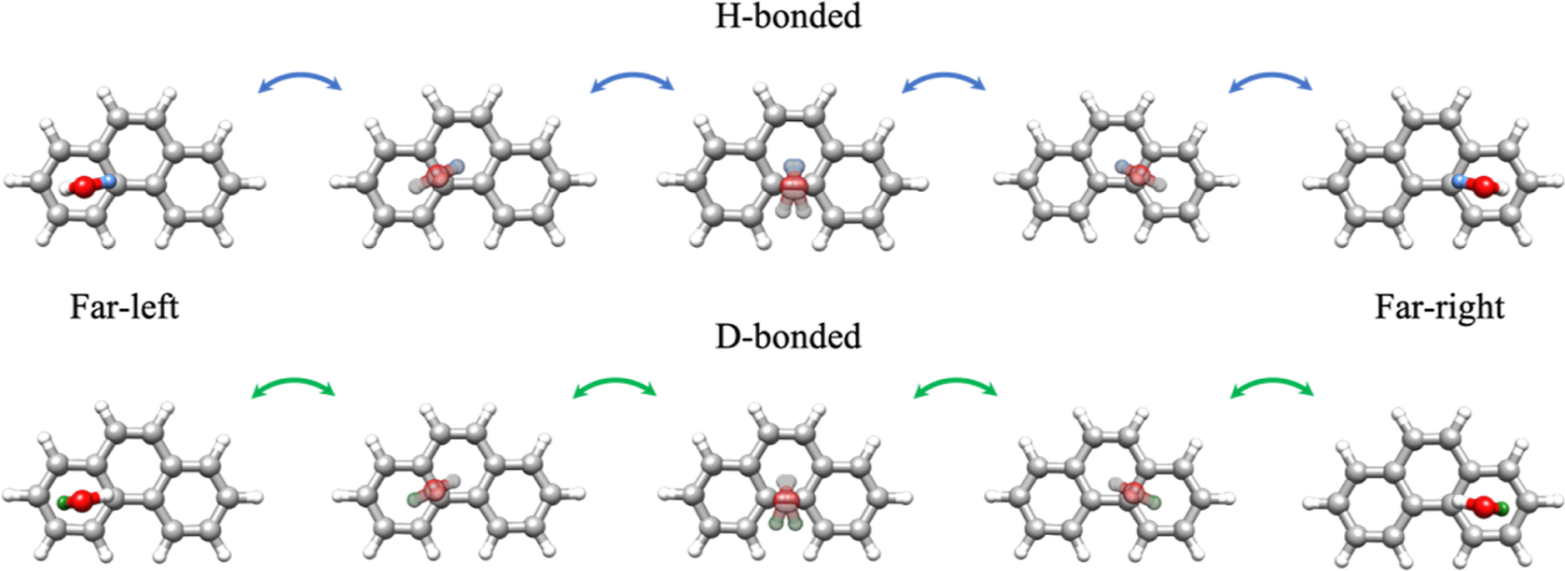
Two pairs of equivalent minima of the Phe–HDO complex. The deuterium atom is indicated as a blue sphere in the case of the H-bonded complex and as a green sphere in the case of the D-bonded complex. The picture is a representation of one of the two possible pathways for the translation of water involving the migration of the water molecule between the two peripheral rings through the middle ring of phenanthrene. The other pathway would involve a migration of water through the bay region of phenanthrene. The pathway was simulated using the NEB method.^35^

This splitting is explained by the existence of two possible pairs of equivalent minima of the monodeuterated complex of phenanthrene. These differ in the position of the deuterium atom: either pointing to a peripheral ring (D-bonded) or to the middle ring (H-bonded) of phenanthrene (Figure 4). A one-dimensional symmetric potential energy path directly connecting the members of each pair of equivalent isomers can be envisioned from a concerted motion in which water translates and rotates simultaneously, as shown in Figure 4. The origin of the corresponding coordinate would be analogous to a configuration similar to the sym-middle geometry in which the entire HDO molecule lies on the σ_*bc*_ plane. This motion preserves the same symmetry properties as in the parent species. Therefore, the same vibration–rotation Hamiltonian used to fit the parent species spectrum can be used for the monodeuterated water complex. The experimental spectroscopic parameters for the monodeuterated complex of phenanthrene are reported in Table S4. The experimental rotational constants are assigned to the D-bonded isomer of the phenanthrene–HDO isotopologue. This assignment was achieved by comparing the experimental rotational constants with the predicted rotational constants for the two isomers of the monodeuterated complex and applying the calculated difference between the theoretical and experimental rotational constants as for the parent species.

The dynamics of the water molecule in the Phe–H_2_O complex is a multidimensional problem. However, to assess the translational minimum energy pathways along the potential energy function describing the dynamics of the water molecule, we used Meyer’s one-dimensional flexible model,^21^ on the basis of the structural relaxation parameters calculated at the B3LYP-D3/6-311++G(d,p) level of theory (Table S5). For the translational motion, the experimental rotational constants and the Δ*E*_0^+^1^+^_ splittings of the isotopic species are reasonably reproduced by the following potential energy function
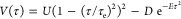
3

**Figure 5:**
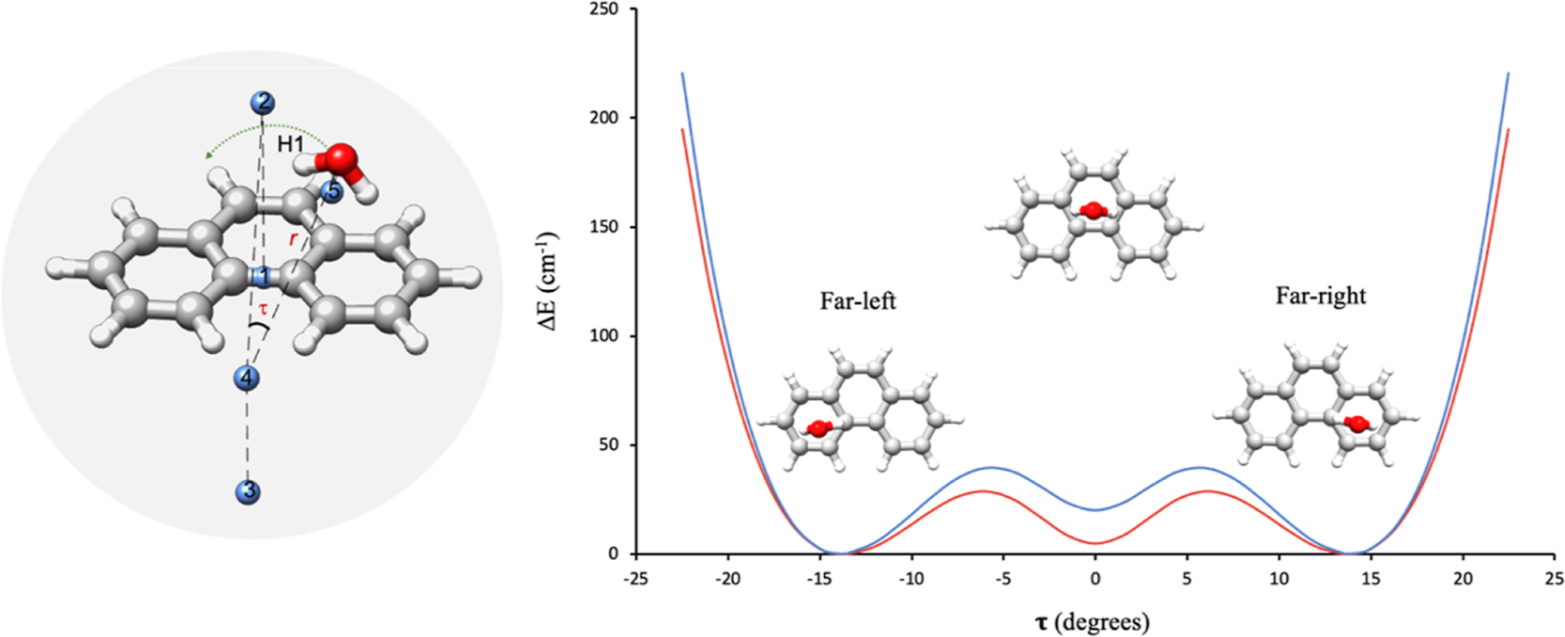
On the right are the predicted (red) and experimental (blue) potential energy curves describing water’s translation motion as a function of the angle τ. On the left, the labeling of the five dummy atoms that are necessary to define this translation correctly is provided.

The first term describes a double minimum well, where *U* is the barrier at τ = 0° and τ_e_ corresponds to the equilibrium values of the angle τ (Figures 5 and S5). In this case, the potential energy function has two equivalent minima at τ_e_ = ±13.7°, which correspond to the two equivalent configurations of the far-isomer (Figure 5). The second term based on the *D* and *E* parameters corrects the shape of this function to give a minimum at the sym-middle configuration (τ = 0°).

Values of *U* = 84.34 cm^–1^, *D* = 64 cm^–1^, and *E* = 125 best reproduce the experimental value of Δ*E*_0^+^1^+^_ for all the water isotopologues of the complex (Table 2).

**Table 2:** Δ*E*_0^+^1^+^_ Values in Megahertz Determined from the Observed Spectral Splitting and from Meyer’s Flexible Model for the Phe–H_2_O, Phe–H_2_^18^O, and Phe–D_2_O Complexes

Δ*E*_0^+^1^+^_ (MHz)	experimental	flexible model
H_2_O	14.0634(77)	14.04
H_2_^18^O	7.4209(65)	7.78
D_2_O	5.0676(66)	7.69

To model the internal rotation of water around its *C*_2_ internal symmetry axis, we considered the angle γ = H_w1_-O-5-4, defined in Figure S5, with the following function
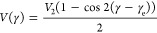
4

**Figure 6:**
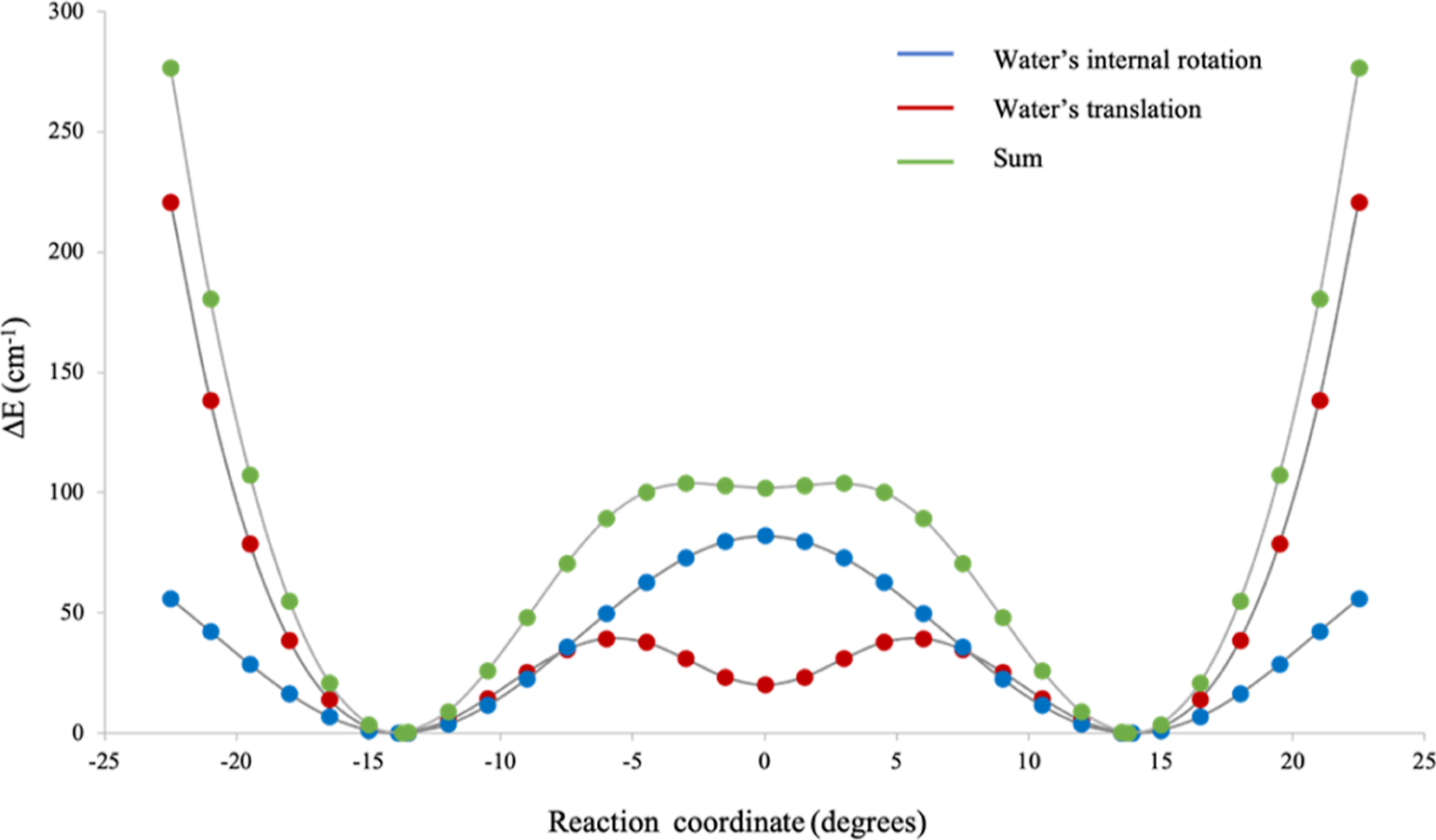
Potential energy curves describing water’s translation motion (red), its internal rotation (blue), and the combination of the two motions (green).

The combination of both motions, translation and internal rotation of the water molecule, was described assuming that the minima at the displacement coordinates τ = ±13.9° are also minima for the internal coordinate γ. As described in Section S7 of the Supporting Information, this makes it possible to assume that the combined translation and internal rotation motions follow a minimum energy path given by a potential energy function obtained by summing the equations describing the independent motions, as depicted in Figure 6.

For *V*_2_ = 86.2 cm^–1^ and γ_e_ = −90°, the values of Δ*E*_0^+^1^–^_ calculated for the two geometries (H-bonded and D-bonded) of the Phe–HDO complex reported in Table 3 are obtained. The best agreement is obtained for the D-bonded geometry of the Phe–HDO complex, in agreement with the structural assignment based on the rotational constants. For the monodeuterated water complex of Phe, the assignment of the coupled states for the translational water’s motion is (0^+^, 1^–^) and (0^–^, 1^+^).

**Table 3:** Δ*E*_0^+^1^–^_ Values Determined from the Observed Spectral Splittings and from Meyer’s Flexible Model for the Phe–HDO Complex

Δ*E*_0^+^1^–^_ (MHz)	experimental
Phe–HDO	0.2464(27)

Δ*E*_0^+^1^–^_ (MHz)	flexible model
H-bonded	0.16
D-bonded	0.27

It is interesting to compare the internal dynamics in the monohydrated complex of phenanthrene with other water complexes in which water couples with an aromatic substrate, such as the benzene–water complex^37^ and the PAH–water complexes acenaphthene–H_2_O,^17^ phenanthridine–H_2_O,^20^ [4]helicene–H_2_O,^38^ and corannulene–H_2_O.^34^ The case of the benzene–water complex is particularly intriguing as even though the aromatic surface is limited to a single benzene ring and the water molecule cannot migrate from one aromatic ring to an equivalent one, the dynamics of water still remain complex due to the small or no-barrier of the internal rotation of the water molecule around the sixfold axis of benzene. A similar case to the benzene–water complex is the monohydrate complex of corannulene. In the corannulene–H_2_O complex, in which dispersion interactions dominate, the water molecule is located inside the bowl-like structure of corannulene, and it is connected to the substrate via two weak O–H···π hydrogen-bond interactions. The rotational spectrum reveals that the water molecule can rotate almost freely about its *C*_2_ axis. In the monohydrated complexes of acenaphthene, [4]-helicene, and phenanthridine, the water molecule forms hydrogen bonds (either as a hydrogen bond acceptor or as a donor) to the aliphatic hydrogen atoms of acenaphthene, the aromatic hydrogen atoms of [4]-helicene, and the nitrogen atom in the phenanthridine backbone, respectively. These interactions constrain the dynamics of water to its internal rotation around its *C*_2_ symmetry axis and do not allow a translation of water on the substrate. In contrast, in the phenanthrene–H_2_O cluster, the symmetry and planarity of the substrate significantly complicate the internal dynamics of the water molecule. Water is no longer limited to its rotation around its symmetry axis, but it is also able to translate above the aromatic surface. Thus, the water molecule moves on the surface of phenanthrene, while in the other systems, water is confined due to substrate modifications. A comparison between these different systems provides insights into how water’s mobility can be altered by modifying the graphene morphology.

## Conclusions

The present study reveals the high mobility of the water molecule when it interacts with a planar carbon surface. A comparison of the internal dynamics of the water molecule in the monohydrated complex of Phe with other previously studied related PAH–water complexes reveals how molecular structure and degree of aromaticity of the substrate influence the behavior of water.

The *C*_2*v*_ symmetry of phenanthrene reduces to *C*_*s*_ or *C*_1_ for the complex. The energetically preferred and experimentally observed *C*_1_ forms have two equivalent configurations, which interconvert through a large amplitude motion in which water moves across the phenanthrene symmetry plane at which the complex adopts a *C*_*s*_ symmetry. The rotational spectrum shows the tunneling splitting arising by this interconversion. Tunneling effects due to water internal rotation have also been observed. The combination of both motions results in a remarkable dynamics, whose detailed analysis (both experimental and theoretical) and interpretation are the main topics of the present article.

A previous study on the motion of water monomers on a graphene surface already revealed that water monomers diffuse continuously prior to ice formation through a motion involving a hopping mechanism from the center of one hexagonal ring to the equivalent one.^9^ By considering the phenanthrene–water complex as a prototypical system for larger planar molecules exhibiting fused six-membered rings, our study provides detailed and crucial information on the migration pathway of the water molecule from one aromatic ring to the other which occurs via a concerted tunneling motion and sets a benchmark for the modeling of single water molecules moving on the surface of extended aromatic systems. The internal dynamics due to quantum tunneling effects observed in the phenanthrene–H_2_O complex might also help to elucidate how water flows through narrow carbon nanotubes, where recent studies showed that the water transport through carbon nanotubes under confined conditions is significantly faster than what current theory of fluid dynamics would predict due to probable quantum effects.^39^ Mechanisms as revealed in the present work on model systems should be considered for this.

## Supporting Information Available

Description of the experimental methods; theoretical spectroscopic parameters of the five unique isomers of the Phe–H_2_O complex calculated at the PBEh-3c level of theory and relative single-point energies calculated at the DLPNO-CCSD(T) level of theory; experimental spectra of Phe–H_2_^16^O, Phe–H_2_^18^O, Phe–D_2_O, and Phe–HDO; experimental spectroscopic parameters of Phe–H_2_^18^O, Phe–D_2_O, and Phe–HDO; description of Meyer’s flexible model; experimental transition frequencies in the unit of megahertz; and the corresponding observed–calculated differences (Δν) in the unit of MHz (PDF)
