# Supplementary material for: Quantum Tunneling Facilitates Water Motion across the Surface of Phenanthrene
Source: J Am Chem Soc. 2023 Jul 26;145(31):17201–10. doi: 10.1021/jacs.3c04281 (PMC10416304; doi:10.1021/jacs.3c04281)
Supplement: Supplementary file 1 — ja3c04281_si_001.pdf [file ja3c04281_si_001.pdf]

Supplementary materials for:

Quantum tunnelling facilitates the water motion across the surface of phenanthrene

Donatella Loru [a], Amanda L. Steber [a] [†], Cristóbal Pérez[a] [†], Daniel A. Obenchain [a] [‡], Berhane Temelso [b], Juan C. López [c] and Melanie Schnell.*[a] [d]

[a] Deutsches Elektronen-Synchrotron DESY, Notkestr. 85, 22607 Hamburg, Germany
[b] Division of Information Technology, College of Charleston, Charleston, SC 29424, USA
[c] Departamento de Química Física y Química Inorgánica, Facultad de Ciencias, Universidad de Valladolid, 47011 Valladolid, Spain
[d] Institut für Physikalische Chemie, Christian-Albrechts-Universität zu Kiel, Max-Eyth-Straße 1, D-24118 Kiel, Germany
[†] Current address: Departamento de Química Física y Química Inorgánica, Facultad de Ciencias, Universidad de Valladolid, 47011 Valladolid, Spain
[‡] Current address: Georg-August-Universität Göttingen, Institut für Physikalische Chemie, 37077 Göttingen, Germany

Table of Contents

S1. Experimental methods **2**
S2. Theoretical results **2**
S3. Phenanthrene – H_2O **3**
S4. Phenanthrene – $H_2{}^{18}O$ **4**
S5. Phenanthrene – D_2O **6**
S6. Phenanthrene – HDO **8**
S7. Meyer's flexible model **10**
S8. List of transitions **15**

S1. Experimental methods

The rotational spectrum of the Phe-H_2O cluster was recorded in the 2-8 GHz frequency range using the CP-FTMW spectrometer COMPACT, which has been reported previously.[1][2] The sample of Phe ($C_{14}H_{10}$, 98% purity) was purchased from Sigma Aldrich and used without any further purification. It appears as a white/yellowish powder with a tabulated melting point of 101°C. To increase the concentration of phenanthrene in the gas phase, the sample was heated to a temperature of ca. 130-135 °C by using a heatable reservoir situated at the orifice of a modified pulsed valve (Parker General Valve, Series 9). The Phe-H_2O complex was formed by introducing water via an external reservoir directly connected to the one containing phenanthrene and positioned inside the vacuum chamber. The Phe-$H_2{}^{18}O$ and Phe-D_2O isotopologues were formed by using an isotopically enriched sample of $H_2{}^{18}O$ and D_2O, respectively. The Phe-HDO complex was formed by using a 1:1 mixture of H_2O and D_2O, since this mixture is known to undergo fast proton exchange.

In the COMPACT spectrometer, the supersonic jet is created by streamlining the carrier gas neon, used at a backing pressure of ca. 3.5 bar, first through the external reservoir containing water and then through the reservoir just close to the valve orifice containing the sample of phenanthrene. Once created, the supersonic jet is probed by a 4 μs long microwave chirped pulse spanning 2–8 GHz, which is generated by an arbitrary waveform generator. Before being broadcasted into the vacuum chamber by a horn antenna, the chirped pulse is amplified by a travelling wave tube amplifier. The interaction of the chirped pulse with the internally cold molecules in the supersonic jet can produce a molecular polarization, which occurs when at least one of the frequencies of the chirped pulse is resonant with a rotational transition of the probed molecules. The polarization is followed by molecular relaxation which produces emission signal. The latter is then recorded by a receiving horn antenna, amplified using a low noise amplifier and digitized in the time domain, using a fast oscilloscope. The signal in the time domain is then converted to the frequency domain by Fast Fourier transformation. The repetition rate of the experiment was set to 8 Hz; in addition, the fast frame mode of the oscilloscope was applied to all the experiments. Thus, each molecular pulse was probed by eight microwave chirped pulses, followed by eight FIDs to be recorded, thus resulting in an effective repetition rate of 64 Hz.

S2. Theoretical results

Table S1 Theoretical rotational constants and dipole moment components of the three unique isomers of the phenanthrene-H_2O complex calculated at the PBEh-3c level of theory and relative single-point energies calculated at the DLPNO-CCSD(T) level of theory.

	far-right/left-1	int-right/left-1	int-right/left-2	sym-middle-1	sym-middle-2
A (MHz)	1080.1	1050.7	1043.5	1052.4	970.6
B (MHz)	449.8	473.2	470.5	472.2	506.0
C (MHz)	394.8	408.1	404.5	412.8	378.5
$\|\mu_a\|$ (D)	1.0	0.1	0.4	0.0	0.0
$\|\mu_b\|$ (D)	0.4	1.0	1.0	0.7	2.1
$\|\mu_c\|$ (D)	2.3	2.2	2.2	2.3	0.4
ΔE_e (kJ/mol)	0.0	0.0	0.4	0.4	0.8

S3. Phenanthrene – H_2O

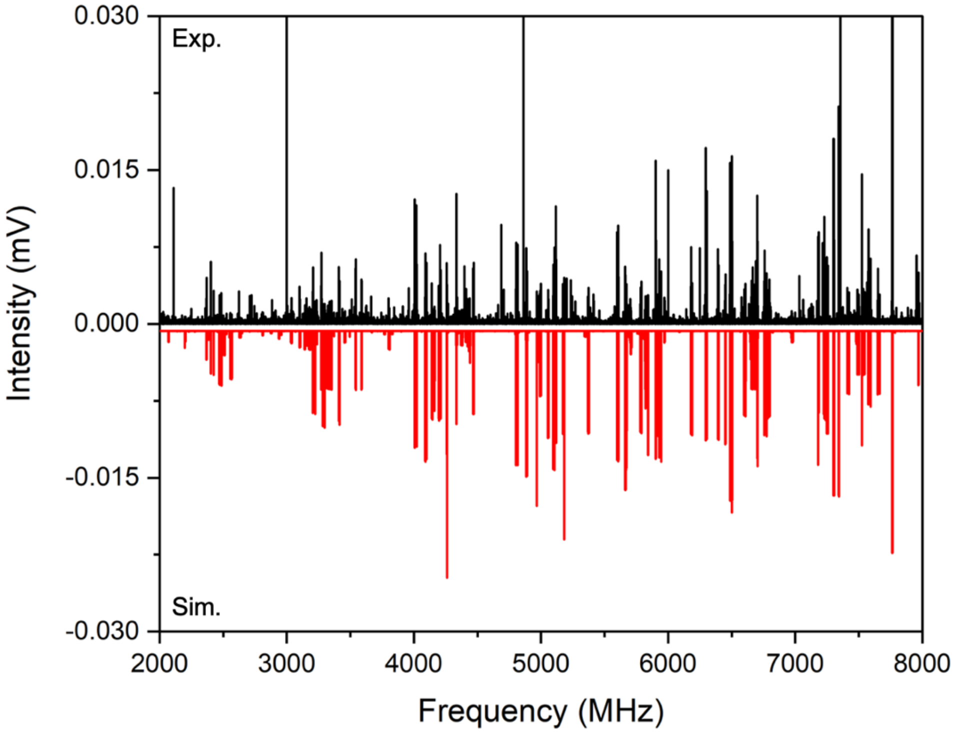

Figure S1 Experimental (black trace) and simulated (red trace) rotational spectrum of the phenanthrene-H_2O complex in the 2-8 GHz frequency range. The simulated spectrum is based on the experimental rotational constants. The experimental spectrum was initially reported in our previous study on the phenanthrene-water clusters.[3]

S4. Phenanthrene – $H_2{}^{18}O$

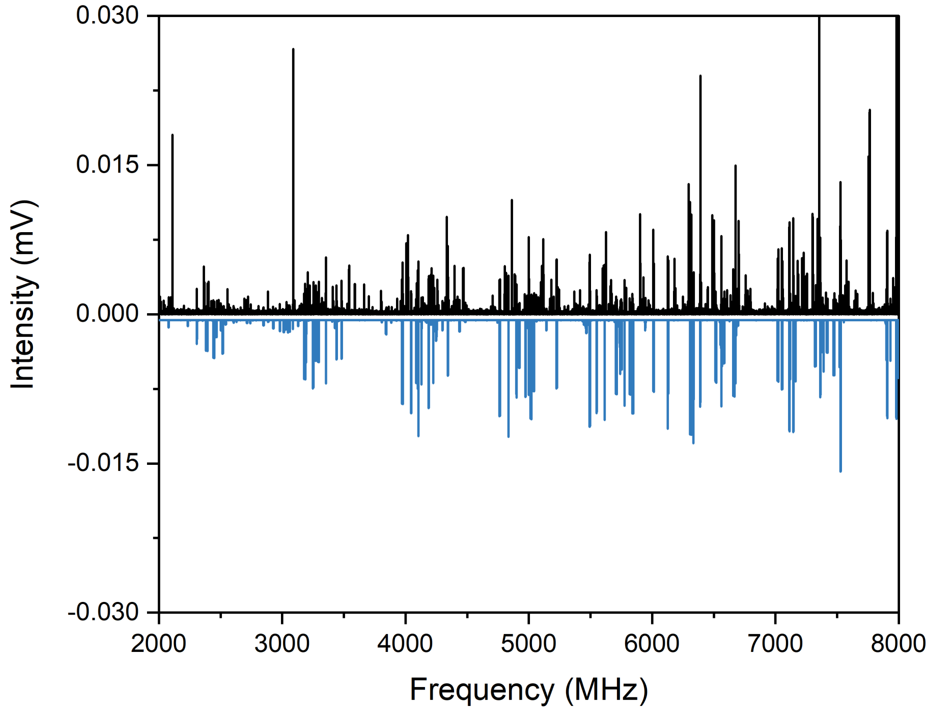

Figure S2 Experimental (black trace) and simulated (blue trace) rotational spectrum of the phenanthrene-$H_2{}^{18}O$ complex in the 2-8 GHz frequency range. The simulated spectrum is based on the experimental rotational constants. The experimental spectrum was initially reported in our previous study on the phenanthrene-water clusters.[3]

Table S2 Experimental spectroscopic constants of the far-left/-right isomer of the phenanthrene-$H_2{}^{18}O$ complex. The experimental rotational transitions were fitted using the two-states coupled rotational Hamiltonian as for the parent species.

	0^+	1^+	0^-	1^-
A (MHz)[a]	1013.434(39)[g]	1013.428(39)	1013.217(35)	1013.211(35)
B (MHz)	431.03802(87)	430.32752(92)	431.06082(68)	430.45643(61)
C (MHz)	391.307(39)	391.296(39)	391.150(35)	391.141(35)
D_J (kHz)[b]	2.421(43)	0.3740(76)	2.094(31)	0.3302(59)
D_K (kHz)	-	-1.383(29)	-	-1.267(24)
D_{JK} (kHz)	-4.77(11)	-1.214(41)	-3.957(77)	-0.909(30)
d_1 (kHz)	-	1.263(24)	-	1.092(17)
d_2 (kHz)	-0.1067(29)	0.1299(35)	-0.0908(22)	0.1169(24)
Δ_{0+1+} (MHz)[c]	7.4209(65)			
Δ_{0-1-} (MHz)	6.3549(52)			
F_b (MHz)[d]	48.75(25)		47.87(22)	
F_{bJ} (MHz)	-0.01686(42)		-0.01472(32)	
N[e]	375			
σ (kHz)[f]	10.4			

[a] *A, B,* and *C* are the rotational constants. [b] D_J, D_K, D_{JK}, d_1, and d_2 are the centrifugal distortion constants. [c] Δ_{0+1+} and Δ_{0-1-} are the differences in vibrational energy between the two tunnelling states 0^+ and 1^+ and 0^- and 1^-, respectively. [d] F_b and F_{bJ} are the Coriolis coupling terms. [e] N is the number of lines in the fit. [f] σ is the root-mean-square deviation of the fit. [g] Standard error within parentheses are expressed in units of the last two digits.

S5. Phenanthrene – D_2O

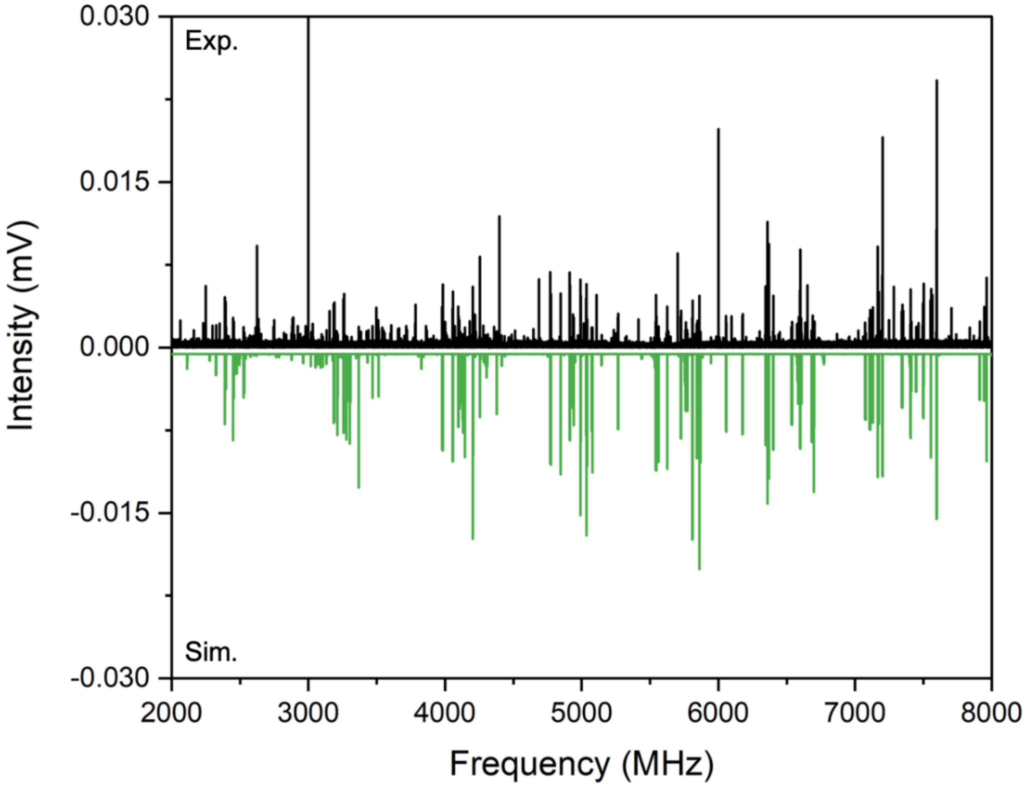

Figure S3 Experimental (black trace) and simulated (green trace) rotational spectrum of the phenanthrene-D_2O complex in the 2-8 GHz frequency range. The simulated spectrum is based on the experimental rotational constants. The experimental spectrum of phenanthrene-D_2O is reported for the first time in this study.

Table S3 Experimental spectroscopic constants of the far-left/-right isomer of the phenanthrene-D_2O complex. The experimental rotational transitions were fitted using the two-states coupled rotational Hamiltonian as for the parent species.

	0^+	1^+	0^-	1^-
A (MHz)[a]	1023.203(70)[g]	1023.194(70)	1023.279(70)	1023.272(70)
B (MHz)	433.15839(73)	432.70053(78)	433.15815(75)	432.68489(79)
C (MHz)	391.260(70)	391.253(70)	391.319(70)	391.312(70)
D_J (kHz) [b]	1.604(40)	0.2952(77)	1.623(42)	0.2719(77)
D_K (kHz)	-	-1.216(74)	-	-1.227(72)
D_{JK} (kHz)	-2.78(11)	-0.510(51)	-2.86(11)	-0.499(49)
d_1 (kHz)	-	0.813(22)	-	0.839(24)
d_2 (kHz)	-0.0657(30)	0.0923(33)	-0.0640(31)	0.0983(34)
Δ_{0+1+} (MHz) [c]	5.0676(66)			
Δ_{0-1-} (MHz)	5.2246(57)			
F_b (MHz) [d]	46.37(47)		46.58(47)	
F_{bJ} (MHz)	-0.01164(45)		-0.01165(47)	
N [e]	248			
σ (kHz)[f]	9.3			

[a]*A, B,* and *C* are the rotational constants. [b]D_J, D_K, D_{JK}, d_1, and d_2 are the centrifugal distortion constants. [c]Δ_{0+1+} and Δ_{0-1-} are the differences in vibrational energy between the two tunnelling states 0^+ and 1^+ and 0^- and 1^-, respectively. [d]F_b and F_{bJ} are the Coriolis coupling terms. [e]N is the number of lines in the fit. [f]σ is the root-mean-square deviation of the fit. [g]Standard error within parentheses are expressed in units of the last two digits.

S6. Phenanthrene – HDO

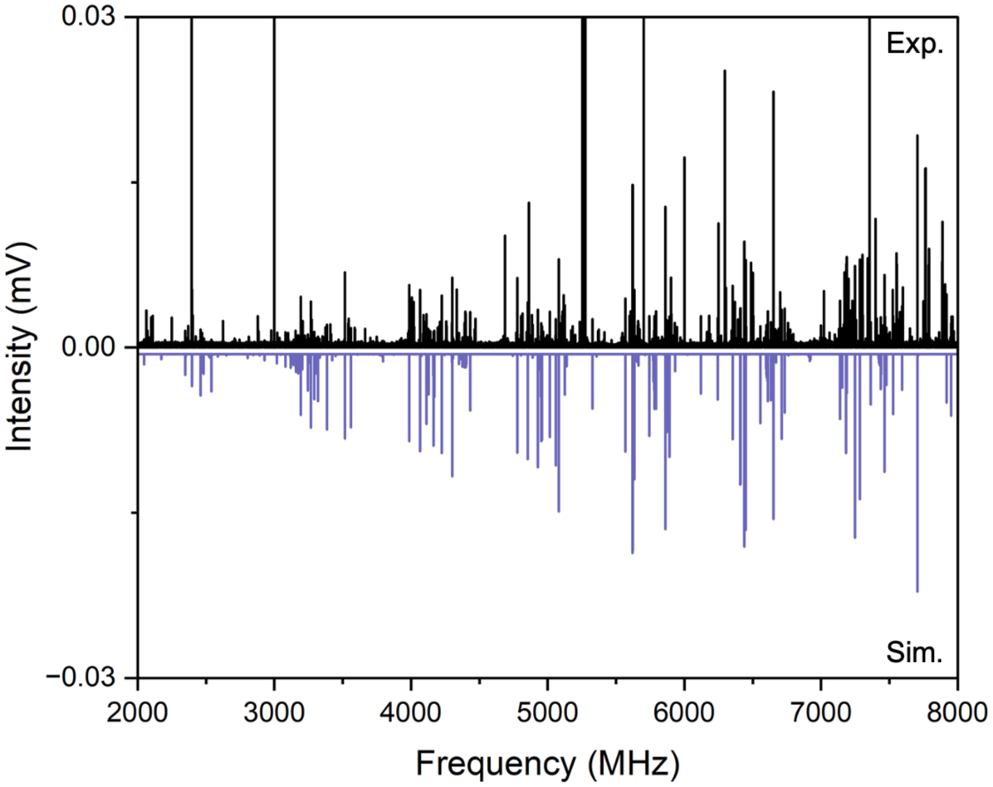

Figure S4 Experimental (black trace) and simulated (violet trace) rotational spectrum of the phenanthrene-HDO complex in the 2-8 GHz frequency range. The simulated spectrum is based on the experimental rotational constants. The experimental spectrum of phenanthrene-HDO is reported for the first time in this study.

Table S4 Experimental spectroscopic constants of the far-left/-right isomer of the phenanthrene-HDO complex. The experimental rotational transitions were fitted using the two-states coupled rotational Hamiltonian as for the parent species.

<table>
<tr><th></th><th>0^+</th><th>1^-</th><th>0^-</th><th>1^+</th></tr>
<tr><td>A (MHz) [a]</td><td>1037.361(47) [g]</td><td>[1037.361] [h]</td><td>1037.365(47)</td><td>[1037.365] [i]</td></tr>
<tr><td>B (MHz)</td><td>435.68171(34)</td><td>435.66391(30)</td><td>435.68597(33)</td><td>435.66749(31)</td></tr>
<tr><td>C (MHz)</td><td>392.253(47)</td><td>[392.253]</td><td>392.251(47)</td><td>[392.251]</td></tr>
<tr><td>D_J (kHz) [b]</td><td colspan="4">0.1045(15)</td></tr>
<tr><td>D_K (kHz)</td><td colspan="4">0.458(27)</td></tr>
<tr><td>D_{JK} (kHz)</td><td>0.8226(75)</td><td>[0.8226]</td><td>0.8225(75)</td><td>[0.8225]</td></tr>
<tr><td>d_1 (kHz)</td><td colspan="4">-0.0081(10)</td></tr>
<tr><td>d_2 (kHz)</td><td colspan="4">0.01543(80)</td></tr>
<tr><td>Δ_{0+1-}(MHz) [c]</td><td colspan="4">0.2464(27)</td></tr>
<tr><td>Δ_{0-1+} (MHz)</td><td colspan="4">0.2297(26)</td></tr>
<tr><td>F_b (MHz) [d]</td><td colspan="2">53.21(28)</td><td colspan="2">53.21(28)</td></tr>
<tr><td>N [e]</td><td colspan="4">406</td></tr>
<tr><td>σ (kHz) [f]</td><td colspan="4">9.8</td></tr>
</table>

[a] *A, B,* and *C* are the rotational constants. [b] D_J, D_K, D_{JK}, d_1, and d_2 are the centrifugal distortion constants. [c] Δ_{0+1+} and Δ_{0-1-} are the differences in vibrational energy between the two tunnelling states 0^+ and 1^+ and 0^- and 1^-, respectively. [d] F_b is the Coriolis coupling terms. [e] N is the number of lines in the fit. [f] σ is the root-mean-square deviation of the fit. [g] Standard error within parentheses are expressed in units of the last two digits. [h] Value fixed to the one of the 0^+ tunnelling state. [i] Value fixed to the one of the 0^- tunnelling state.

S7. Meyer's flexible model

The water's internal dynamics observed in the phenanthrene-H_2O complex has been assessed by applying the Meyer's flexible model, which is generally used to determine the potential energy surface from rotational and vibrational experimental data[4]. This model describes the intramolecular motion as a function of one structural parameter selected as an independent variable. The rest of the parameters, instead of remaining fixed, can vary as a function of the independent variable.

In the case of the phenanthrene-water complex, we selected the angle τ as the independent variable to describe the translational motion of the water molecule with respect to the substrate phenanthrene (Figure S5). To correctly describe the motion as a function of τ, we defined five dummy atoms as illustrated in Figure S7.

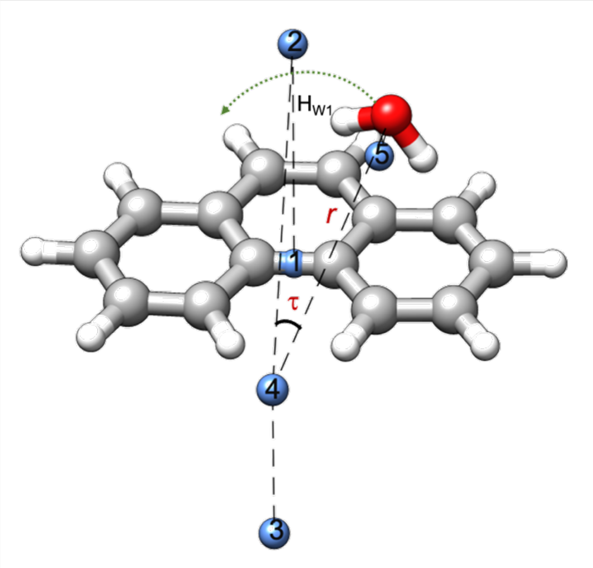

Figure S5 Schematic representation of the dummy atoms defined to describe the translation of the water molecule in the phenanthrene-H_2O complex.

The one-dimensional potential energy function describing the tunnelling of the water molecule between the left and right configuration of the **far**-isomer as a function of the angle τ was explored at the B3LYP-D3/6-311++G(2d,p) level of theory, and it was fitted to the following expression:

$$V(\tau) = U(1-(\tau/\tau_e)^2)^2 - De^{-E\tau^2} \quad (1)$$

where U corresponds to the potential energy barrier at τ = 0° and τ_e corresponds to the equilibrium values of the angle τ. The second term is based on the D and E parameters, it corrects the shape of the function to give a minimum corresponding to the sym-middle geometry of the complex (τ = 0°).

Equation 1 was used in combination with the following relaxation parameters as predicted by calculations performed at the B3LYP-D3/6-311G(d,p) level of theory: the distance r = O-4, the angle β = 5-O-4, the dihedral ε = 5-O-4-3, and the dihedral γ = H_{w1}-O-5-4, where the numbers denote the dummy atoms.

$$r = r_0 + r(1)e^{-r(2)\tau^2} - r(3)\tau^2 \quad (2)$$

$$\beta(°) = 91.44 - 2.5e^{-0.025\tau^2} - 0.0144\tau^2 \quad (3)$$

$$\varepsilon = -0.3759\tau - 0.0024\tau^3 \quad (4)$$

$$\gamma = -90.0 - 1.59\tau - 45.1\left(\left(1/(1+e^{-\tau})\right) - 0.5\right) \quad (5)$$

The values considered for all the parameters defined above are reported in Table S5.

Table S5 Values of the parameters utilized to describe the translation of the water molecule in the monohydrated complex of phenanthrene.

	**B3LYP-D3/6-311++G(2dp)**	**Flexible model**
τ_e	±13.7°	13.73°
**B**	69 cm^{-1}	84.34 cm^{-1}
**C**	64 cm^{-1}	64 cm^{-1}
**D**	125	125
***r*(0)**	7.199 Å	7.2476
***r*(1)**	-0.0423 Å	0.0
***r*(2)**	-0.025	0.0
***r*(3)**	-0.00011 Å	0.0

When using the values of the selected relaxation parameters reported in Table S5 in Meyer's one-dimensional model, the experimental values of the rotational constants and of ΔE_{0+1+} are reproduced to a reasonable level as shown in Table S6.

Table S6 Values of the rotational constants A, B and C in MHz and energy differences ΔE_{0+1+} (in MHz) obtained both experimentally and from the flexible model for the water's translation motion relative to the phenanthrene substrate.

	Experimental			Flexible model		
**Parent**	**0^+**	**1^+**	**Δ**	**0^+**	**1^+**	**Δ**
A	1046.730(25)	1046.720(25)	-0.010	1043.935	1043.935	0.000
B	440.2228(11)	439.1122(10)	-1.111	442.115	441.185	-0.935
C	393.590(25)	393.577(25)	-0.013	395.805	395.805	0.000
ΔE_{0+1+}	14.0634(77)			14.04		
**^{18}O**	**0^+**	**1^+**	**Δ**	**0^+**	**1^+**	**Δ**
A	1013.434(39)	1013.428(39)	-0.005	1008.930	1008.943	-0.013
B	431.03802(87)	430.32753(92)	-0.710	432.900	432.265	-0.635
C	391.307(39)	391.296(39)	-0.011	393.610	393.605	-0.005
ΔE_{0+1+}	7.4209(65)			7.78		
**D_2O**	**0^+**	**1^+**	**Δ**	**0^+**	**1^+**	**Δ**
A	1023.203(70)	1023.194(70)	-0.009	1019.965	1019.965	0.000
B	433.15839(73)	432.70053(78)	-0.458	435.215	434.635	-0.580
C	391.260(70)	391.253(70)	-0.007	393.685	393.685	0.000
ΔE_{0+1+}	5.0676(66)			7.69		
**HDO**	**0^+**	**1^-**				
A	1037.361(47)	1037.361(47)	-			
B	435.68171(34)	435.66391(30)				
C	392.253(47)	392.253(47)	-			
ΔE_{0+1-}	0.2464(27)					

To describe the internal rotation of the water around the C_2 internal symmetry axis, we consider the dihedral angle γ as the independent variable. The one-dimensional potential energy function describing the water's internal rotation was fitted to the following function:

$$V(\gamma) = \frac{V_2(1 - cos2(\gamma - \gamma_e))}{2} \qquad (6)$$

Considering a value of V_2 = 86.2 cm^{-1} and γ_e = -90°, the values of ΔE_{0-0+} reported in Table S7 are obtained.

Table S7 Values of the rotational constants A, B and C in MHz and of $\Delta E_{0\text{-}0+}$ obtained both experimentally and from the Meyer's flexible model for the water's internal rotation motion.

	exp			Flexible model		
Parent	0^+	0^-	Δ	0^+	0^-	Δ
A	1046.730(25)	1046.475(28)	-0.255	1045.175	1045.310	0.135
B	440.2228(11)	440.2110(10)	-0.0118	439.551	339.400	-0.115
C	393.590(25)	393.467(28)	-0.123	393.565	393.270	-0.295
$\Delta E_{0\text{-}0+}$	-			89701.4		

The combination of both translational motion and internal rotation of the water molecule was described by considering that the minimum at the vibrational coordinate at τ = 13.7 ° is also a minimum for the internal coordinate γ. This is true if:

$$\gamma - \gamma_e = \left(\frac{180}{2\tau_e}\right)(\tau - \tau_e) \qquad (7)$$

and

$$\tau = \tau_e + \left(\frac{2\tau_e}{180}\right)(\gamma - \gamma_e) \qquad (8)$$

In this case, the potential energy function describing the combination of the two motions can be expressed as the sum of the potential energy function describing the translation of the water (equation 1) and the one describing the internal rotation of the water (equation 6).

$$V(\tau) = U(1 - (\frac{\tau}{\tau_e})^2)^2 - De^{-E\tau^2} + V_2(1 - cos2\left(\left(\frac{180}{2\tau_e}\right)(\tau - \tau_e)\right))/2 \qquad (9)$$

Considering the values obtained from the flexible model in Table S5, V_2 = 86.2 cm^{-1} and γ_e = -90º, we have calculated the values of the rotational constants and ΔE for the monodeuterated complex of phenanthrene (Table S8).

Table S8 Values of the rotational constants A, B and C in MHz and energy differences $\Delta E_{0+1-/0-1+}$ (in MHz) obtained both experimentally and from the flexible model for the water's concerted motion observed in the Phe-HDO complex.

	Experimental		
	0^+	1^-	Δ
A (MHz)	1037.361(47)	1037.361(47)	0.00
B (MHz)	435.68171(34)	435.66391(30)	0.02
C (MHz)	392.253(47)	392.253(47)	0.00
ΔE (MHz)	0.2464(27)		
D-Bonded	Flexible model		
	v=0	v=1	Δ
A (MHz)	1036.16	1036.16	0.00
B (MHz)	436.55	436.55	0.00
C (MHz)	392.83	392.83	0.00
ΔE (MHz)	0.270		
H- Bonded	Flexible model		
	v=0	v=1	Δ
A (MHz)	1031.51	1031.505	0.00
B (MHz)	437.48	437.485	0.00
C (MHz)	394.23	394.235	0.00
ΔE (MHz)	0.16		

S8. List of transitions

The fitted rotational transitions have been grouped together in the following manner: first, transitions belonging to the *R*-branch and *Q*-branch have been separated. Within each branch, the *a*-type transitions are separated from the *c*-type transitions. Furthermore, we have sorted the transitions based on the lower and upper tunnelling states, namely 0^+, 1^+, 0^-, and 1^-.

Table S9 Measured frequencies (ν_{obs}) in MHz and residuals ($\nu_{obs} - \nu_{calc}$) in MHz of the rotational transitions of the parent species of Phe-H_2O.

^{a} *R*-branch										
J	*K_a*	*K_c*	v	←	*J′*	*K_a′*	*K_c′*	v′	ν_{obs}	ν_{obs}-ν_{calc}
				$0^+ \leftarrow 1^+$						
3	1	3	0^+	←	2	1	2	1^+	2401.5502	-0.0035
3	1	2	0^+	←	2	1	1	1^+	2554.8125	0.0079
4	1	4	0^+	←	3	1	3	1^+	3204.6926	0.0013
4	0	4	0^+	←	3	0	3	1^+	3284.2429	0.0084
4	1	3	0^+	←	3	1	2	1^+	3409.7907	0.0014
5	1	5	0^+	←	4	1	4	1^+	4005.5354	0.0026
5	0	5	0^+	←	4	0	4	1^+	4089.5481	0.0070
5	2	4	0^+	←	4	2	3	1^+	4139.3245	0.0119
5	2	3	0^+	←	4	2	2	1^+	4195.6539	-0.0100
5	1	4	0^+	←	4	1	3	1^+	4261.2365	-0.0143
6	1	6	0^+	←	5	1	5	1^+	4803.6288	-0.0099
6	0	6	0^+	←	5	0	5	1^+	4884.1406	-0.0035
6	2	5	0^+	←	5	2	4	1^+	4966.3393	0.0272
6	4	3	0^+	←	5	4	2	1^+	4984.8217	0.0050
6	4	2	0^+	←	5	4	1	1^+	4984.9397	0.0079
6	3	4	0^+	←	5	3	3	1^+	4990.9996	0.0121
6	3	3	0^+	←	5	3	2	1^+	4996.8645	0.0229
6	2	4	0^+	←	5	2	3	1^+	5059.9681	-0.0231
6	1	5	0^+	←	5	1	4	1^+	5107.2389	-0.0266
7	1	7	0^+	←	6	1	6	1^+	5598.7863	-0.0184
7	0	7	0^+	←	6	0	6	1^+	5669.5930	0.0003
7	2	6	0^+	←	6	2	5	1^+	5789.8671	-0.0187
7	5	3	0^+	←	6	5	2	1^+	5816.3506	0.0160
7	5	2	0^+	←	6	5	1	1^+	5816.3506	0.0160
7	3	5	0^+	←	6	3	4	1^+	5829.1789	0.0000
7	3	4	0^+	←	6	3	3	1^+	5842.1028	0.0058
7	2	5	0^+	←	6	2	4	1^+	5928.8243	-0.0256
7	1	6	0^+	←	6	1	5	1^+	5945.8111	-0.0166
8	1	8	0^+	←	7	1	7	1^+	6391.0362	-0.0202
8	0	8	0^+	←	7	0	7	1^+	6448.8116	-0.0068
8	4	5	0^+	←	7	4	4	1^+	6662.0243	0.0107
8	4	4	0^+	←	7	4	3	1^+	6663.0661	0.0133
8	3	5	0^+	←	7	3	4	1^+	6691.5133	0.0206
8	1	7	0^+	←	7	1	6	1^+	6775.0888	0.0115
8	2	6	0^+	←	7	2	5	1^+	6798.2548	0.0062
9	1	9	0^+	←	8	1	8	1^+	7180.6072	-0.0027
9	0	9	0^+	←	8	0	8	1^+	7224.9317	0.0171
9	3	6	0^+	←	8	3	5	1^+	7546.0243	-0.0093
3	1	2	0^+	←	2	1	1	1^+	2554.8131	0.0085
5	3	3	0^+	←	4	3	2	1^+	4152.6264	-0.0008
5	3	2	0^+	←	4	3	1	1^+	4154.8483	-0.0003
8	6	3	0^+	←	7	6	2	1^+	6647.7839	-0.0078
8	6	2	0^+	←	7	6	1	1^+	6647.7839	-0.0078
				$1^+ \leftarrow 0^+$						
3	1	3	1^+	←	2	1	2	0^+	2425.4369	0.0309
3	0	3	1^+	←	2	0	2	0^+	2487.3128	0.0023
4	1	4	1^+	←	3	1	3	0^+	3224.9955	0.0109

J	K_a	K_c	v	←	J'	K_a'	K_c'	v′	ν_{obs}	ν_{obs}-ν_{calc}
4	0	4	1^+	←	3	0	3	0^+	3297.7667	0.0117
4	1	3	1^+	←	3	1	2	0^+	3414.5864	-0.0141
5	1	5	1^+	←	4	1	4	0^+	4022.0811	0.0037
5	0	5	1^+	←	4	0	4	0^+	4098.6760	0.0207
5	2	4	1^+	←	4	2	3	0^+	4145.9712	-0.0591
5	2	3	1^+	←	4	2	2	0^+	4199.7235	0.0061
5	1	4	1^+	←	4	1	3	0^+	4256.8936	-0.0012
6	1	6	1^+	←	5	1	5	0^+	4816.6048	-0.0090
6	0	6	1^+	←	5	0	5	0^+	4890.1362	0.0171
6	2	5	1^+	←	5	2	4	0^+	4965.5115	-0.0213
6	3	4	1^+	←	5	3	3	0^+	4992.8514	-0.0108
6	4	3	1^+	←	5	4	2	0^+	4992.2804	0.0140
6	4	2	1^+	←	5	4	1	0^+	4992.4005	0.0191
6	3	3	1^+	←	5	3	2	0^+	4998.6421	-0.0225
6	2	4	1^+	←	5	2	3	0^+	5055.2630	-0.0021
6	1	5	1^+	←	5	1	4	0^+	5095.2534	0.0137
7	1	7	1^+	←	6	1	6	0^+	5608.6359	-0.0137
7	0	7	1^+	←	6	0	6	0^+	5673.7250	0.0045
7	2	6	1^+	←	6	2	5	0^+	5782.7537	-0.0153
7	2	5	1^+	←	6	2	4	0^+	5917.2469	-0.0040
8	1	8	1^+	←	7	1	7	0^+	6398.3300	-0.0123
8	0	8	1^+	←	7	0	7	0^+	6452.0154	-0.0128
8	2	7	1^+	←	7	2	6	0^+	6597.5752	-0.0030
8	3	6	1^+	←	7	3	5	0^+	6655.7352	0.0158
8	4	4	1^+	←	7	4	3	0^+	6655.2916	-0.0138
8	3	5	1^+	←	7	3	4	0^+	6681.1006	-0.0164
8	1	7	1^+	←	7	1	6	0^+	6755.5783	0.0015
8	2	6	1^+	←	7	2	5	0^+	6782.8948	-0.0126
9	1	9	1^+	←	8	1	8	0^+	7185.9326	0.0112
9	0	9	1^+	←	8	0	8	0^+	7227.6738	-0.0019
9	2	8	1^+	←	8	2	7	0^+	7409.7345	0.0226
9	2	7	1^+	←	8	2	6	0^+	7648.8844	0.0016
5	3	3	1^+	←	4	3	2	0^+	4163.2108	0.0138
5	3	2	1^+	←	4	3	1	0^+	4165.4010	0.0163
7	3	5	1^+	←	6	3	4	0^+	5823.7262	-0.0091
8	6	3	1^+	←	7	6	2	0^+	6649.9981	0.0135
8	6	2	1^+	←	7	6	1	0^+	6649.9981	0.0135
					**0^- ← 1^-**					
3	1	3	0^-	←	2	1	2	1^-	2403.1977	-0.0056
3	0	3	0^-	←	2	0	2	1^-	2470.0496	-0.0012
3	1	2	0^-	←	2	1	1	1^-	2556.0491	0.0121
4	1	4	0^-	←	3	1	3	1^-	3206.0386	0.0003
4	0	4	0^-	←	3	0	3	1^-	3285.1849	0.0096
4	1	3	0^-	←	3	1	2	1^-	3410.4020	0.0061
5	1	5	0^-	←	4	1	4	1^-	4006.5580	0.0043
5	0	5	0^-	←	4	0	4	1^-	4090.0801	0.0070
5	2	4	0^-	←	4	2	3	1^-	4139.9013	0.0176
5	2	3	0^-	←	4	2	2	1^-	4196.3037	-0.0109
5	1	4	0^-	←	4	1	3	1^-	4261.2365	-0.0143
6	1	6	0^-	←	5	1	5	1^-	4804.3339	-0.0048
6	0	6	0^-	←	5	0	5	1^-	4884.3330	-0.0008
6	2	5	0^-	←	5	2	4	1^-	4966.3393	0.0272
6	4	3	0^-	←	5	4	2	1^-	4985.5817	0.0077
6	4	2	0^-	←	5	4	1	1^-	4985.6951	0.0048
6	3	4	0^-	←	5	3	3	1^-	4991.3537	0.0114
6	2	4	0^-	←	5	2	3	1^-	5060.1439	-0.0258
6	1	5	0^-	←	5	1	4	1^-	5106.7084	-0.0210
7	1	7	0^-	←	6	1	6	1^-	5599.1975	-0.0120
7	0	7	0^-	←	6	0	6	1^-	5669.5241	-0.0018

J	K_a	K_c	v	←	J'	K_a'	K_c'	v′	ν_{obs}	ν_{obs}-ν_{calc}
7	2	6	0^-	←	6	2	5	1^-	5789.4340	-0.0040
7	5	3	0^-	←	6	5	2	1^-	5816.9454	0.0149
7	5	2	0^-	←	6	5	1	1^-	5816.9454	0.0149
7	3	5	0^-	←	6	3	4	1^-	5829.0617	0.0008
7	3	4	0^-	←	6	3	3	1^-	5842.1028	0.0058
7	1	6	0^-	←	6	1	5	1^-	5944.9122	-0.0154
8	1	8	0^-	←	7	1	7	1^-	6391.1900	-0.0147
8	0	8	0^-	←	7	0	7	1^-	6448.5718	-0.0020
8	2	7	0^-	←	7	2	6	1^-	6608.6031	-0.0174
8	6	3	0^-	←	7	6	2	1^-	6648.2486	0.0015
8	6	2	0^-	←	7	6	1	1^-	6648.2486	0.0015
8	4	5	0^-	←	7	4	4	1^-	6661.8104	0.0083
8	4	4	0^-	←	7	4	3	1^-	6662.8595	0.0062
8	3	5	0^-	←	7	3	4	1^-	6691.3004	0.0164
8	1	7	0^-	←	7	1	6	1^-	6773.9943	0.0149
8	2	6	0^-	←	7	2	5	1^-	6797.9964	0.0002
9	1	9	0^-	←	8	1	8	1^-	7180.5448	-0.0009
9	0	9	0^-	←	8	0	8	1^-	7224.5644	0.0158
9	3	6	0^-	←	8	3	5	1^-	7545.8777	-0.0012
4	2	3	0^-	←	3	2	2	1^-	3310.8743	0.0043
5	3	3	0^-	←	4	3	2	1^-	4153.5629	-0.0039
5	3	2	0^-	←	4	3	1	1^-	4155.8139	0.0100
					**1^-← 0^-**					
3	1	3	1^-	←	2	1	2	0^-	2423.6695	0.0229
3	0	3	1^-	←	2	0	2	0^-	2486.0584	-0.0013
3	1	2	1^-	←	2	1	1	0^-	2568.2318	-0.0049
4	1	4	1^-	←	3	1	3	0^-	3223.4691	0.0126
4	0	4	1^-	←	3	0	3	0^-	3296.8408	-0.0013
4	1	3	1^-	←	3	1	2	0^-	3414.6495	-0.0101
5	1	5	1^-	←	4	1	4	0^-	4020.7829	0.0023
5	0	5	1^-	←	4	0	4	0^-	4097.9924	0.0102
5	2	4	1^-	←	4	2	3	0^-	4145.7452	-0.0190
5	2	3	1^-	←	4	2	2	0^-	4199.8914	0.0051
5	1	4	1^-	←	4	1	3	0^-	4257.6714	-0.0032
6	1	6	1^-	←	5	1	5	0^-	4815.5140	-0.0066
6	0	6	1^-	←	5	0	5	0^-	4889.5691	0.0135
6	2	5	1^-	←	5	2	4	0^-	4965.7970	-0.0201
6	3	4	1^-	←	5	3	3	0^-	4993.0566	-0.0069
6	4	3	1^-	←	5	4	2	0^-	4992.0640	0.0100
6	4	2	1^-	←	5	4	1	0^-	4992.1824	0.0122
6	3	3	1^-	←	5	3	2	0^-	4998.8931	-0.0189
6	2	4	1^-	←	5	2	3	0^-	5056.2175	0.0062
6	1	5	1^-	←	5	1	4	0^-	5096.6026	0.0135
7	1	7	1^-	←	6	1	6	0^-	5607.7050	-0.0110
7	0	7	1^-	←	6	0	6	0^-	5673.1624	0.0050
7	2	6	1^-	←	6	2	5	0^-	5783.4838	-0.0147
7	3	4	1^-	←	6	3	3	0^-	5837.5045	-0.0205
7	2	5	1^-	←	6	2	4	0^-	5918.8208	0.0054
7	1	6	1^-	←	6	1	5	0^-	5930.3386	0.0182
8	1	8	1^-	←	7	1	7	0^-	6397.5103	-0.0090
8	0	8	1^-	←	7	0	7	0^-	6451.3976	-0.0097
8	2	7	1^-	←	7	2	6	0^-	6598.6079	-0.0084
8	4	4	1^-	←	7	4	3	0^-	6656.2954	-0.0077
8	3	6	1^-	←	7	3	5	0^-	6656.9103	0.0180
8	4	5	1^-	←	7	4	4	0^-	6655.2438	-0.0027
8	3	5	1^-	←	7	3	4	0^-	6682.4383	-0.0122
8	1	7	1^-	←	7	1	6	0^-	6757.3558	0.0023
8	2	6	1^-	←	7	2	5	0^-	6784.8262	-0.0027
9	1	9	1^-	←	8	1	8	0^-	7185.1695	0.0036

J	K_a	K_c	v	←	J'	K_a'	K_c'	v′	ν_{obs}	ν_{obs}-ν_{calc}
9	0	9	1^-	←	8	0	8	0^-	7226.9821	-0.0060
9	2	8	1^-	←	8	2	7	0^-	7410.9261	0.0104
9	6	4	1^-	←	8	6	3	0^-	7479.8987	0.0123
9	6	3	1^-	←	8	6	2	0^-	7479.8987	0.0123
9	3	6	1^-	←	8	3	5	0^-	7535.2605	0.0207
9	2	7	1^-	←	8	2	6	0^-	7650.8594	-0.0062
4	2	3	1^-	←	3	2	2	0^-	3323.4824	0.0096
4	2	2	1^-	←	3	2	1	0^-	3351.3614	0.0149
5	3	3	1^-	←	4	3	2	0^-	4162.7252	0.0122
5	3	2	1^-	←	4	3	1	0^-	4164.9262	0.0068
7	3	5	1^-	←	6	3	4	0^-	5824.5003	-0.0060
					**^{c}*R*-branch**					
					**$0^+ \leftarrow 0^+$**					
6	1	6	0^+	←	5	2	4	0^+	2521.0109	-0.0119
7	1	7	0^+	←	6	2	5	0^+	3157.7837	0.0031
2	2	0	0^+	←	1	1	0	0^+	3543.5248	-0.0154
2	2	1	0^+	←	1	1	1	0^+	3590.7118	-0.0061
6	0	6	0^+	←	5	1	4	0^+	3801.3381	-0.0007
3	2	1	0^+	←	2	1	1	0^+	4335.4836	-0.0092
7	1	6	0^+	←	6	2	4	0^+	4338.2339	-0.0026
7	0	7	0^+	←	6	1	5	0^+	4370.4117	0.0034
3	2	2	0^+	←	2	1	2	0^+	4471.1470	0.0093
8	0	8	0^+	←	7	1	6	0^+	4882.8106	0.0002
4	2	2	0^+	←	3	1	2	0^+	5118.9749	-0.0059
5	1	4	0^+	←	4	0	4	0^+	5181.4579	0.0016
4	2	3	0^+	←	3	1	3	0^+	5375.8254	0.0009
3	3	0	0^+	←	2	2	0	0^+	5664.8410	-0.0228
3	3	1	0^+	←	2	2	1	0^+	5667.6674	-0.0249
5	2	3	0^+	←	4	1	3	0^+	5904.4030	-0.0085
9	1	8	0^+	←	8	2	6	0^+	5971.8025	0.0104
6	1	5	0^+	←	5	0	5	0^+	6189.2965	-0.0068
5	2	4	0^+	←	4	1	4	0^+	6304.6969	0.0035
4	3	1	0^+	←	3	2	1	0^+	6489.6212	0.0030
4	3	2	0^+	←	3	2	2	0^+	6503.4598	0.0022
6	2	4	0^+	←	5	1	4	0^+	6702.8039	-0.0030
7	1	6	0^+	←	6	0	6	0^+	7239.7229	-0.0057
6	2	5	0^+	←	5	1	5	0^+	7257.6211	-0.0168
5	3	2	0^+	←	4	2	2	0^+	7304.4371	0.0234
5	3	3	0^+	←	4	2	3	0^+	7344.6654	0.0131
7	2	5	0^+	←	6	1	5	0^+	7524.3625	-0.0036
					**$1^+ \leftarrow 1^+$**					
6	1	6	1^+	←	5	2	4	1^+	2525.2759	-0.0004
7	1	7	1^+	←	6	2	5	1^+	3164.1310	-0.0087
2	2	0	1^+	←	1	1	0	1^+	3543.4227	0.0164
2	2	1	1^+	←	1	1	1	1^+	3589.4731	0.0182
6	0	6	1^+	←	5	1	4	1^+	3808.0771	-0.0042
7	1	6	1^+	←	6	2	4	1^+	4333.1013	-0.0113
3	2	1	1^+	←	2	1	1	1^+	4335.0641	0.0064
9	1	9	1^+	←	8	2	7	1^+	4353.6553	0.0018
3	2	2	1^+	←	2	1	2	1^+	4467.6573	0.0041
4	2	2	1^+	←	3	1	2	1^+	5118.5442	0.0072
5	1	4	1^+	←	4	0	4	1^+	5171.6015	0.0228
4	2	3	1^+	←	3	1	3	1^+	5370.0619	-0.0101
9	0	9	1^+	←	8	1	7	1^+	5354.9104	0.0010
3	3	0	1^+	←	2	2	0	1^+	5664.2951	0.0243
3	3	1	1^+	←	2	2	1	1^+	5667.0297	0.0179
5	2	3	1^+	←	4	1	3	1^+	5904.0999	0.0022
9	1	8	1^+	←	8	2	6	1^+	5970.6917	0.0141
6	1	5	1^+	←	5	0	5	1^+	6178.0569	0.0118

J	K_a	K_c	v	←	J'	K_a'	K_c'	v′	ν_{obs}	ν_{obs}-ν_{calc}
5	2	4	1^+	←	4	1	4	1^+	6296.8536	-0.0165
4	3	1	1^+	←	3	2	1	1^+	6488.2996	0.0067
4	3	2	1^+	←	3	2	2	1^+	6501.7648	0.0148
6	2	4	1^+	←	5	1	4	1^+	6702.8039	-0.0030
7	1	6	1^+	←	6	0	6	1^+	7227.8095	-0.0036
6	2	5	1^+	←	5	1	5	1^+	7248.1287	-0.0200
5	3	2	1^+	←	4	2	2	1^+	7303.0196	0.0017
5	3	3	1^+	←	4	2	3	1^+	7342.3232	0.0092
					**0^- ← 0^-**					
6	1	6	0^-	←	5	2	4	0^-	2521.3906	0.0025
7	1	7	0^-	←	6	2	5	0^-	3157.8755	-0.0061
5	0	5	0^-	←	4	1	3	0^-	3174.7063	-0.0164
6	1	5	0^-	←	5	2	3	0^-	3460.9637	-0.0098
2	2	0	0^-	←	1	1	0	0^-	3542.4644	-0.0177
2	2	1	0^-	←	1	1	1	0^-	3589.6584	-0.0059
6	0	6	0^-	←	5	1	4	0^-	3800.8660	0.0019
3	2	1	0^-	←	2	1	1	0^-	4334.3911	-0.0109
7	1	6	0^-	←	6	2	4	0^-	4339.4629	0.0043
7	0	7	0^-	←	6	1	5	0^-	4369.4042	0.0040
3	2	2	0^-	←	2	1	2	0^-	4470.0648	0.0050
9	2	7	0^-	←	8	3	5	0^-	4802.8254	0.0063
10	2	9	0^-	←	9	3	7	0^-	4825.7536	0.0077
8	0	8	0^-	←	7	1	6	0^-	4881.0798	0.0012
4	2	2	0^-	←	3	1	2	0^-	5117.8956	-0.0097
5	1	4	0^-	←	4	0	4	0^-	5181.4579	0.0016
9	0	9	0^-	←	8	1	7	0^-	5341.0471	-0.0092
4	2	3	0^-	←	3	1	3	0^-	5374.7796	0.0086
3	3	0	0^-	←	2	2	0	0^-	5663.0943	-0.0207
10	0	10	0^-	←	9	1	8	0^-	5758.1278	0.0023
5	2	3	0^-	←	4	1	3	0^-	5903.4147	-0.0158
6	1	5	0^-	←	5	0	5	0^-	6189.6719	-0.0094
5	2	4	0^-	←	4	1	4	0^-	6303.7352	0.0038
4	3	1	0^-	←	3	2	1	0^-	6487.8235	-0.0046
4	3	2	0^-	←	3	2	2	0^-	6501.7025	-0.0027
6	2	4	0^-	←	5	1	4	0^-	6702.0493	-0.0089
10	1	9	0^-	←	9	2	7	0^-	6706.1321	-0.0084
7	1	6	0^-	←	6	0	6	0^-	7240.6472	-0.0054
6	2	5	0^-	←	5	1	5	0^-	7256.8364	-0.0120
5	3	2	0^-	←	4	2	2	0^-	7302.6053	0.0155
5	3	3	0^-	←	4	2	3	0^-	7342.9506	0.0035
7	2	5	0^-	←	6	1	5	0^-	7523.9376	-0.0078
					**1^- ← 1^-**					
6	1	6	1^-	←	5	2	4	1^-	22525.2759	0.0130
7	1	7	1^-	←	6	2	5	1^-	3163.2915	0.0045
5	0	5	1^-	←	4	1	3	1^-	3177.7714	-0.0023
6	1	5	1^-	←	5	2	3	1^-	3454.6936	-0.0070
2	2	0	1^-	←	1	1	0	1^-	3542.3816	0.0145
2	2	1	1^-	←	1	1	1	1^-	3588.6037	0.0095
6	0	6	1^-	←	5	1	4	1^-	3806.6007	-0.0029
3	2	1	1^-	←	2	1	1	1^-	4334.0323	0.0059
9	1	9	1^-	←	8	2	7	1^-	4351.6109	0.0074
3	2	2	1^-	←	2	1	2	1^-	4467.1017	-0.0025
9	2	7	1^-	←	8	3	5	1^-	4801.6653	-0.0243
4	2	2	1^-	←	3	1	2	1^-	5117.5177	0.0059
5	1	4	1^-	←	4	0	4	1^-	5173.0425	0.0175
8	1	7	1^-	←	7	2	5	1^-	5177.9982	0.0015
9	0	9	1^-	←	8	1	7	1^-	5350.7154	0.0021
4	2	3	1^-	←	3	1	3	1^-	5369.8701	-0.0158
3	3	0	1^-	←	2	2	0	1^-	5662.6325	0.0202

J	K_a	K_c	v	←	J'	K_a'	K_c'	v′	ν_{obs}	ν_{obs}-ν_{calc}
3	3	1	1^-	←	2	2	1	1^-	5665.3902	0.0172
5	2	3	1^-	←	4	1	3	1^-	5903.1341	0.0021
9	1	8	1^-	←	8	2	6	1^-	5971.4096	0.0087
6	1	5	1^-	←	5	0	5	1^-	6180.0720	0.0131
5	2	4	1^-	←	4	1	4	1^-	6297.0620	-0.0166
4	3	1	1^-	←	3	2	1	1^-	6486.7018	0.0000
4	3	2	1^-	←	3	2	2	1^-	6500.2575	0.0072
6	2	4	1^-	←	5	1	4	1^-	6701.9691	0.0019
7	1	6	1^-	←	6	0	6	1^-	7230.4469	0.0007
6	2	5	1^-	←	5	1	5	1^-	7248.7512	-0.0166
5	3	2	1^-	←	4	2	2	1^-	7301.3989	-0.0021
5	3	3	1^-	←	4	2	3	1^-	7340.9479	0.0024
7	2	5	1^-	←	6	1	5	1^-	7524.2682	-0.0163
					**^{c}*Q*-branch**					
					**$0^+ \leftarrow 0^+$**					
4	3	2	0^+	←	4	2	2	0^+	3141.2484	-0.0085
5	3	2	0^+	←	5	2	4	0^+	3202.9609	-0.0003
6	3	3	0^+	←	6	2	5	0^+	3235.1181	0.0288
9	4	6	0^+	←	9	3	6	0^+	4336.9751	-0.0328
8	4	5	0^+	←	8	3	5	0^+	4381.6874	0.0005
7	4	4	0^+	←	7	3	4	0^+	4409.4637	0.0044
6	4	3	0^+	←	6	3	3	0^+	4425.7179	0.0060
9	4	5	0^+	←	9	3	7	0^+	4432.4451	0.0138
6	4	2	0^+	←	6	3	4	0^+	4434.6534	0.0125
9	5	4	0^+	←	9	4	6	0^+	5690.4610	-0.0136
7	5	3	0^+	←	7	4	3	0^+	5702.6195	-0.0158
7	5	2	0^+	←	7	4	4	0^+	5703.1508	-0.0152
					**$1^+ \leftarrow 1^+$**					
6	3	4	1^+	←	6	2	4	1^+	3039.0401	0.0154
5	3	2	1^+	←	5	2	4	1^+	3204.5517	0.0034
6	3	3	1^+	←	6	2	5	1^+	3236.1019	0.0088
7	3	4	1^+	←	7	2	6	1^+	3289.0077	0.0264
9	4	6	1^+	←	9	3	6	1^+	4337.0755	0.0283
8	4	5	1^+	←	8	3	5	1^+	4382.6204	0.0183
7	4	4	1^+	←	7	3	4	1^+	4411.1646	-0.0013
6	4	3	1^+	←	6	3	3	1^+	4428.1626	0.0015
7	4	3	1^+	←	7	3	5	1^+	4433.3955	-0.0073
6	4	2	1^+	←	6	3	4	1^+	4437.0576	-0.0046
9	5	4	1^+	←	9	4	6	1^+	5690.9892	0.0012
8	5	3	1^+	←	8	4	5	1^+	5699.2288	-0.0178
7	5	3	1^+	←	7	4	3	1^+	5705.0381	-0.0232
7	5	2	1^+	←	7	4	4	1^+	5705.5742	-0.0187
					**$0^- \leftarrow 0^-$**					
5	3	3	0^-	←	5	2	3	0^-	3099.5254	0.0153
4	3	2	0^-	←	4	2	2	0^-	3139.5113	-0.0097
4	3	1	0^-	←	4	2	3	0^-	3183.5869	-0.0171
5	3	2	0^-	←	5	2	4	0^-	3201.4140	-0.0034
6	3	3	0^-	←	6	2	5	0^-	3233.6872	0.0107
8	2	6	0^-	←	8	1	8	0^-	3355.3977	0.0113
8	4	5	0^-	←	8	3	5	0^-	4378.8167	-0.0044
7	4	4	0^-	←	7	3	4	0^-	4406.8331	0.0037
6	4	3	0^-	←	6	3	3	0^-	4423.2415	0.0019
6	4	2	0^-	←	6	3	4	0^-	4432.2409	0.0023
9	5	4	0^-	←	9	4	6	0^-	5687.1107	-0.0051
8	5	4	0^-	←	8	4	4	0^-	5692.9731	0.0036
8	5	3	0^-	←	8	4	5	0^-	5694.5885	0.0105
7	5	3	0^-	←	7	4	3	0^-	5699.4627	-0.0030
7	5	2	0^-	←	7	4	4	0^-	5700.0022	-0.0001
9	6	4	0^-	←	9	5	4	0^-	6965.6744	-0.0112

J	K_a	K_c	v	←	J'	K_a'	K_c'	v′	ν_{obs}	ν_{obs}-ν_{calc}
9	6	3	0^-	←	9	5	5	0^-	6965.7598	-0.0168
					$1^- \leftarrow 1^-$					
6	3	4	1^-	←	6	2	4	1^-	3036.3636	0.0012
5	3	3	1^-	←	5	2	3	1^-	3102.3497	0.0018
4	3	2	1^-	←	4	2	2	1^-	3142.2672	0.0093
4	3	1	1^-	←	4	2	3	1^-	3185.5118	0.0144
5	3	2	1^-	←	5	2	4	1^-	3202.7603	0.0010
6	3	3	1^-	←	6	2	5	1^-	3234.5127	0.0003
7	3	4	1^-	←	7	2	6	1^-	3287.7115	0.0087
8	4	5	1^-	←	8	3	5	1^-	4379.6429	0.0176
7	4	4	1^-	←	7	3	4	1^-	4408.2969	-0.0060
6	4	3	1^-	←	6	3	3	1^-	4425.3377	-0.0006
8	4	4	1^-	←	8	3	6	1^-	4428.6356	0.0087
7	4	3	1^-	←	7	3	5	1^-	4430.7075	0.0006
6	4	2	1^-	←	6	3	4	1^-	4434.3026	-0.0067
5	4	2	1^-	←	5	3	2	1^-	4434.9203	0.0138
5	4	1	1^-	←	5	3	3	1^-	4437.9057	0.0151
9	5	4	1^-	←	9	4	6	1^-	5687.5574	-0.0111
7	5	3	1^-	←	7	4	3	1^-	5701.5310	-0.0164
7	5	2	1^-	←	7	4	4	1^-	5702.0714	-0.0134
6	5	1	1^-	←	6	4	3	1^-	5706.6574	0.0057
6	5	2	1^-	←	6	4	2	1^-	5706.5152	0.0099

Table S10 Measured frequencies (ν_{obs}) in MHz and residuals ($\nu_{obs} - \nu_{calc}$) in MHz of the rotational transitions of the parent species of Phe-$H_2{}^{18}O$.

					***a* R-branch**					
J	K_a	K_c	v	←	J'	K_a'	K_c'	v′	ν_{obs}	ν_{obs}-ν_{calc}
					$0^+ \leftarrow 1^+$					
3	0	3	0^+	←	2	0	2	1^+	2440.9848	0.0028
3	1	2	0^+	←	2	1	1	1^+	2514.7981	0.0214
4	1	4	0^+	←	3	1	3	1^+	3177.0547	0.0004
4	0	4	0^+	←	3	0	3	1^+	3247.2968	0.0114
4	2	3	0^+	←	3	2	2	1^+	3267.7019	0.0017
4	2	2	0^+	←	3	2	1	1^+	3290.5811	-0.0077
4	1	3	0^+	←	3	1	2	1^+	3353.9765	0.0090
5	1	5	0^+	←	4	1	4	1^+	3969.8804	0.0000
5	0	5	0^+	←	4	0	4	1^+	4045.2622	0.0043
5	2	4	0^+	←	4	2	3	1^+	4084.9128	0.0156
5	3	2	0^+	←	4	3	1	1^+	4097.4688	0.0037
5	2	3	0^+	←	4	2	2	1^+	4129.5140	-0.0017
5	1	4	0^+	←	4	1	3	1^+	4190.2546	-0.0035
6	1	6	0^+	←	5	1	5	1^+	4760.4366	-0.0107
6	0	6	0^+	←	5	0	5	1^+	4834.3405	-0.0071
6	2	5	0^+	←	5	2	4	1^+	4899.9343	-0.0036
6	4	3	0^+	←	5	4	2	1^+	4915.4891	0.0079
6	4	2	0^+	←	5	4	1	1^+	4915.5598	0.0024
6	3	4	0^+	←	5	3	3	1^+	4919.9110	-0.0037
6	3	3	0^+	←	5	3	2	1^+	4924.1613	0.0237
6	2	4	0^+	←	5	2	3	1^+	4974.6069	-0.0110
6	1	5	0^+	←	5	1	4	1^+	5022.2194	-0.0124
7	1	7	0^+	←	6	1	6	1^+	5548.5649	-0.0121
7	0	7	0^+	←	6	0	6	1^+	5615.3237	-0.0089
7	2	6	0^+	←	6	2	5	1^+	5712.2170	-0.0128
7	4	3	0^+	←	6	4	2	1^+	5739.7654	0.0156
7	3	5	0^+	←	6	3	4	1^+	5743.8483	-0.0136
7	3	4	0^+	←	6	3	3	1^+	5753.2244	0.0027
7	2	5	0^+	←	6	2	4	1^+	5824.0901	-0.0040

J	K_a	K_c	v	←	J'	K_a'	K_c'	v′	ν_{obs}	ν_{obs}-ν_{calc}
7	1	6	0^+	←	6	1	5	1^+	5848.4701	0.0051
8	1	8	0^+	←	7	1	7	1^+	6334.2832	0.0026
8	0	8	0^+	←	7	0	7	1^+	6390.4814	-0.0001
8	2	7	0^+	←	7	2	6	1^+	6521.3033	-0.0100
8	3	6	0^+	←	7	3	5	1^+	6567.1834	-0.0071
8	3	5	0^+	←	7	3	4	1^+	6585.4895	0.0014
9	1	9	0^+	←	8	1	8	1^+	7117.6687	-0.0105
9	0	9	0^+	←	8	0	8	1^+	7162.2702	0.0032
9	2	8	0^+	←	8	2	7	1^+	7326.8946	-0.0015
10	0	10	0^+	←	9	0	9	1^+	7932.7989	0.0184
					**$1^+ \leftarrow 0^+$**					
3	1	3	1^+	←	2	1	2	0^+	2394.4627	0.0241
4	1	4	1^+	←	3	1	3	0^+	3186.9457	0.0104
4	0	4	1^+	←	3	0	3	0^+	3252.9968	0.0073
4	2	3	1^+	←	3	2	2	0^+	3274.1085	-0.0025
4	2	2	1^+	←	3	2	1	0^+	3296.4201	0.0133
4	1	3	1^+	←	3	1	2	0^+	3354.4981	-0.0096
5	1	5	1^+	←	4	1	4	0^+	3977.4601	0.0009
5	0	5	1^+	←	4	0	4	0^+	4048.3446	0.0126
5	2	4	1^+	←	4	2	3	0^+	4086.5828	-0.0107
5	2	3	1^+	←	4	2	2	0^+	4130.1641	0.0084
5	1	4	1^+	←	4	1	3	0^+	4185.6267	0.0047
6	1	6	1^+	←	5	1	5	0^+	4765.8966	-0.0057
6	0	6	1^+	←	5	0	5	0^+	4835.6272	0.0092
6	2	5	1^+	←	5	2	4	0^+	4897.3984	-0.0116
6	4	2	1^+	←	5	4	1	0^+	4917.9105	0.0055
6	3	3	1^+	←	5	3	2	0^+	4923.3871	-0.0133
6	2	4	1^+	←	5	2	3	0^+	4970.7972	0.0114
6	1	5	1^+	←	5	1	4	0^+	5013.7282	0.0133
7	1	7	1^+	←	6	1	6	0^+	5552.2492	-0.0053
7	0	7	1^+	←	6	0	6	0^+	5615.7554	-0.0050
7	2	6	1^+	←	6	2	5	0^+	5706.4506	0.0107
7	3	5	1^+	←	6	3	4	0^+	5739.4293	-0.0048
7	3	4	1^+	←	6	3	3	0^+	5748.8920	-0.0077
7	2	5	1^+	←	6	2	4	0^+	5817.4063	0.0051
7	1	6	1^+	←	6	1	5	0^+	5837.9247	0.0026
8	1	8	1^+	←	7	1	7	0^+	6336.5751	-0.0143
8	0	8	1^+	←	7	0	7	0^+	6390.6066	-0.0207
8	2	7	1^+	←	7	2	6	0^+	6513.4821	-0.0034
8	4	5	1^+	←	7	4	4	0^+	6558.2387	0.0030
8	4	4	1^+	←	7	4	3	0^+	6558.9461	0.0069
8	3	6	1^+	←	7	3	5	0^+	6560.7186	0.0214
8	3	5	1^+	←	7	3	4	0^+	6579.3270	0.0019
8	2	6	1^+	←	7	2	5	0^+	6667.7865	-0.0253
9	1	9	1^+	←	8	1	8	0^+	7119.0409	-0.0061
9	0	9	1^+	←	8	0	8	0^+	7162.3854	-0.0221
9	2	7	1^+	←	8	2	6	0^+	7519.0799	0.0048
4	4	0	1^+	←	3	3	0	0^+	7529.7090	0.0064
10	1	10	1^+	←	9	1	9	0^+	7899.8312	0.0167
10	0	10	1^+	←	9	0	9	0^+	7932.9652	0.0175
					**$0^- \leftarrow 1^-$**					
3	1	3	0^-	←	2	1	2	1^-	2383.1898	-0.0080
3	0	3	0^-	←	2	0	2	1^-	2441.7149	0.0008
3	1	2	0^-	←	2	1	1	1^-	2515.5103	0.0196
4	1	4	0^-	←	3	1	3	1^-	3177.7433	-0.0068
4	0	4	0^-	←	3	0	3	1^-	3247.7771	0.0051
4	2	3	0^-	←	3	2	2	1^-	3268.3522	0.0045
4	2	2	0^-	←	3	2	1	1^-	3291.3099	-0.0014
4	1	3	0^-	←	3	1	2	1^-	3354.3674	0.0110

J	K_a	K_c	v	←	J'	K_a'	K_c'	v′	ν_{obs}	ν_{obs}-ν_{calc}
5	1	5	0^-	←	4	1	4	1^-	3970.3877	0.0013
5	0	5	0^-	←	4	0	4	1^-	4045.5137	0.0084
5	2	4	0^-	←	4	2	3	1^-	4085.2469	0.0131
5	3	3	0^-	←	4	3	2	1^-	4096.4439	-0.0043
5	3	2	0^-	←	4	3	1	1^-	4098.0642	0.0032
5	1	4	0^-	←	4	1	3	1^-	4190.3487	0.0017
6	1	6	0^-	←	5	1	5	1^-	4760.7698	-0.0002
6	0	6	0^-	←	5	0	5	1^-	4834.3405	-0.0071
6	2	5	0^-	←	5	2	4	1^-	4900.0044	0.0066
6	4	3	0^-	←	5	4	2	1^-	4915.9950	0.0055
6	4	2	0^-	←	5	4	1	1^-	4916.0742	0.0076
6	3	4	0^-	←	5	3	3	1^-	4920.2117	-0.0001
6	3	3	0^-	←	5	3	2	1^-	4924.4856	0.0136
6	2	4	0^-	←	5	2	3	1^-	4974.9438	-0.0129
6	1	5	0^-	←	5	1	4	1^-	5022.0919	-0.0155
7	1	7	0^-	←	6	1	6	1^-	5548.7315	-0.0044
7	0	7	0^-	←	6	0	6	1^-	5615.2216	-0.0063
7	2	6	0^-	←	6	2	5	1^-	5712.0674	-0.0126
7	5	2	0^-	←	6	5	3	1^-	5735.0751	0.0055
7	5	3	0^-	←	6	5	2	1^-	5735.0751	0.0055
7	4	4	0^-	←	6	4	3	1^-	5739.7654	0.0156
7	4	3	0^-	←	6	4	2	1^-	5740.0134	0.0134
7	3	5	0^-	←	6	3	4	1^-	5743.9603	-0.0134
7	3	4	0^-	←	6	3	3	1^-	5753.4212	0.0011
7	2	5	0^-	←	6	2	4	1^-	5824.4085	-0.0088
7	1	6	0^-	←	6	1	5	1^-	5848.2350	-0.0091
8	1	8	0^-	←	7	1	7	1^-	6334.2832	0.0026
8	0	8	0^-	←	7	0	7	1^-	6390.2729	-0.0009
8	2	7	0^-	←	7	2	6	1^-	6521.0193	-0.0177
8	5	3	0^-	←	7	5	2	1^-	6558.2387	0.0030
8	5	4	0^-	←	7	5	3	1^-	6558.2387	0.0030
8	4	5	0^-	←	7	4	4	1^-	6563.6911	-0.0120
8	4	4	0^-	←	7	4	3	1^-	6564.3883	-0.0169
8	3	6	0^-	←	7	3	5	1^-	6567.1834	-0.0071
8	3	5	0^-	←	7	3	4	1^-	6585.7075	-0.0066
8	1	7	0^-	←	7	1	6	1^-	6667.4251	0.0162
8	2	6	0^-	←	7	2	5	1^-	6675.6483	0.0151
9	1	9	0^-	←	8	1	8	1^-	7117.5855	-0.0063
9	0	9	0^-	←	8	0	8	1^-	7161.9965	0.0050
9	2	8	0^-	←	8	2	7	1^-	7326.5588	-0.0141
9	6	3	0^-	←	8	6	2	1^-	7376.7573	-0.0057
9	2	7	0^-	←	8	2	6	1^-	7525.7225	0.0079
10	1	10	0^-	←	9	1	9	1^-	7898.9037	0.0071
10	0	10	0^-	←	9	0	9	1^-	7932.4787	0.0197
					$1^- \leftarrow 0^-$					
3	1	3	1^-	←	2	1	2	0^-	2393.6155	0.0253
3	0	3	1^-	←	2	0	2	0^-	2449.3408	0.0046
3	1	2	1^-	←	2	1	1	0^-	2520.7830	-0.0031
4	1	4	1^-	←	3	1	3	0^-	3186.2574	0.0118
4	0	4	1^-	←	3	0	3	0^-	3252.7199	0.0040
4	2	3	1^-	←	3	2	2	0^-	3273.8943	0.0096
4	2	2	1^-	←	3	2	1	0^-	3296.3438	0.0098
4	1	3	1^-	←	3	1	2	0^-	3354.8895	-0.0092
5	1	5	1^-	←	4	1	4	0^-	3976.9229	0.0013
5	0	5	1^-	←	4	0	4	0^-	4048.2090	0.0075
5	2	4	1^-	←	4	2	3	0^-	4086.7451	-0.0080
5	3	3	1^-	←	4	3	2	0^-	4100.0277	-0.0009
5	3	2	1^-	←	4	3	1	0^-	4101.6529	0.0130
5	1	4	1^-	←	4	1	3	0^-	4186.4588	0.0016

J	K_a	K_c	v	←	J'	K_a'	K_c'	v′	ν_{obs}	ν_{obs}-ν_{calc}
6	1	6	1^-	←	5	1	5	0^-	4765.4857	-0.0056
6	0	6	1^-	←	5	0	5	0^-	4835.5394	0.0071
6	2	5	1^-	←	5	2	4	0^-	4897.8929	-0.0094
6	4	3	1^-	←	5	4	2	0^-	4918.0593	-0.0002
6	4	2	1^-	←	5	4	1	0^-	4918.1426	0.0064
6	3	4	1^-	←	5	3	3	0^-	4919.6118	-0.0047
6	3	3	1^-	←	5	3	2	0^-	4923.8821	-0.0101
6	2	4	1^-	←	5	2	3	0^-	4971.7085	0.0061
6	1	5	1^-	←	5	1	4	0^-	5014.8782	0.0102
7	1	7	1^-	←	6	1	6	0^-	5551.9283	-0.0052
7	0	7	1^-	←	6	0	6	0^-	5615.6371	-0.0016
7	2	6	1^-	←	6	2	5	0^-	5707.1853	0.0095
7	5	3	1^-	←	6	5	2	0^-	5735.8336	-0.0090
7	5	2	1^-	←	6	5	1	0^-	5735.8336	-0.0090
7	4	4	1^-	←	6	4	3	0^-	5737.7646	-0.0102
7	4	3	1^-	←	6	4	2	0^-	5738.0107	-0.0185
7	3	5	1^-	←	6	3	4	0^-	5740.2213	0.0012
7	3	4	1^-	←	6	3	3	0^-	5749.7424	-0.0085
7	2	5	1^-	←	6	2	4	0^-	5818.6732	0.0084
7	1	6	1^-	←	6	1	5	0^-	5839.2281	0.0069
8	1	8	1^-	←	7	1	7	0^-	6336.3103	-0.0090
8	0	8	1^-	←	7	0	7	0^-	6390.4125	-0.0156
8	2	7	1^-	←	7	2	6	0^-	6514.3611	0.0025
8	4	5	1^-	←	7	4	4	0^-	6559.1837	0.0051
8	4	4	1^-	←	7	4	3	0^-	6559.8833	0.0104
8	3	6	1^-	←	7	3	5	0^-	6561.6950	0.0145
8	3	5	1^-	←	7	3	4	0^-	6580.4332	0.0044
8	1	7	1^-	←	7	1	6	0^-	6658.2222	-0.0215
8	2	6	1^-	←	7	2	5	0^-	6669.2254	-0.0162
9	1	9	1^-	←	8	1	8	0^-	7118.7893	-0.0049
9	2	8	1^-	←	8	2	7	0^-	7319.1665	-0.0090
9	6	4	1^-	←	8	6	3	0^-	7372.8700	0.0022
9	6	3	1^-	←	8	6	2	0^-	7372.8700	0.0022
9	2	7	1^-	←	8	2	6	0^-	7520.4962	0.0038
10	1	10	1^-	←	9	1	9	0^-	7899.5698	0.0137
10	0	10	1^-	←	9	0	9	0^-	7932.6284	0.0186
9	0	9	1^-	←	8	0	8	0^-	7162.1121	-0.0165
					**^{c}*R*-branch**					
					**$0^+ \leftarrow 0^+$**					
3	1	2	0^+	←	2	0	2	0^+	3194.8346	0.0229
7	1	7	0^+	←	6	2	5	0^+	3256.7617	-0.0238
6	1	5	0^+	←	5	2	3	0^+	3431.0757	-0.0096
2	2	0	0^+	←	1	1	0	0^+	3441.4925	-0.0153
2	2	1	0^+	←	1	1	1	0^+	3482.7497	-0.0029
6	0	6	0^+	←	5	1	4	0^+	3840.1839	0.0114
4	1	3	0^+	←	3	0	3	0^+	4104.2460	0.0111
3	2	1	0^+	←	2	1	1	0^+	4225.7046	-0.0023
7	4	4	0^+	←	7	3	4	0^+	4228.5135	-0.0099
7	4	3	0^+	←	7	3	5	0^+	4244.5978	-0.0118
3	2	2	0^+	←	2	1	2	0^+	4344.7864	0.0053
4	2	2	0^+	←	3	1	2	0^+	5001.3081	-0.0027
5	1	4	0^+	←	4	0	4	0^+	5042.4470	-0.0141
4	2	3	0^+	←	3	1	3	0^+	5227.9861	0.0081
3	3	0	0^+	←	2	2	0	0^+	5494.5389	-0.0166
3	3	1	0^+	←	2	2	1	0^+	5496.7793	-0.0184
5	2	3	0^+	←	4	1	3	0^+	5776.7585	-0.0099
9	1	8	0^+	←	8	2	6	0^+	5943.8299	0.0129
6	1	5	0^+	←	5	0	5	0^+	6013.8335	-0.0065
5	2	4	0^+	←	4	1	4	0^+	6132.2991	0.0025

J	K_a	K_c	v	←	J'	K_a'	K_c'	v′	ν_{obs}	ν_{obs}-ν_{calc}
4	3	1	0^+	←	3	2	1	0^+	6308.5947	-0.0025
4	3	2	0^+	←	3	2	2	0^+	6319.5927	-0.0039
6	2	4	0^+	←	5	1	4	0^+	6561.3217	0.0032
7	1	6	0^+	←	6	0	6	0^+	7022.0208	0.0135
6	2	5	0^+	←	5	1	5	0^+	7057.6834	-0.0104
5	3	2	0^+	←	4	2	2	0^+	7114.6498	0.0065
5	3	3	0^+	←	4	2	3	0^+	7146.7527	-0.0013
7	2	5	0^+	←	6	1	5	0^+	7363.8115	0.0169
6	3	3	0^+	←	5	2	3	0^+	7908.4134	0.0095
6	3	4	0^+	←	5	2	4	0^+	7980.4922	-0.0037
					**$1^+ \leftarrow 1^+$**					
2	1	1	1^+	←	1	0	1	1^+	2308.2053	0.0105
3	1	2	1^+	←	2	0	2	1^+	3191.2530	0.0049
7	1	7	1^+	←	6	2	5	1^+	3260.4520	-0.0024
6	1	5	1^+	←	5	2	3	1^+	3426.9997	-0.0145
2	2	0	1^+	←	1	1	0	1^+	3441.4575	0.0212
2	2	1	1^+	←	1	1	1	1^+	3481.9551	0.0014
4	1	3	1^+	←	3	0	3	1^+	4099.4943	0.0060
3	2	1	1^+	←	2	1	1	1^+	4225.5023	0.0037
7	1	6	1^+	←	6	2	4	1^+	4298.2203	-0.0013
3	2	2	1^+	←	2	1	2	1^+	4342.5931	-0.0095
4	2	2	1^+	←	3	1	2	1^+	5001.2294	0.0092
5	1	4	1^+	←	4	0	4	1^+	5036.8704	0.0044
4	2	3	1^+	←	3	1	3	1^+	5224.4363	-0.0173
3	3	0	1^+	←	2	2	0	1^+	5494.1906	0.0110
3	3	1	1^+	←	2	2	1	1^+	5496.3903	0.0107
5	2	3	1^+	←	4	1	3	1^+	5776.9627	0.0040
6	1	5	1^+	←	5	0	5	1^+	6007.8442	0.0001
4	3	1	1^+	←	3	2	1	1^+	6307.7651	-0.0021
4	3	2	1^+	←	3	2	2	1^+	6318.5900	0.0016
6	2	4	1^+	←	5	1	4	1^+	6561.9340	0.0017
7	1	6	1^+	←	6	0	6	1^+	7016.1289	-0.0152
6	2	5	1^+	←	5	1	5	1^+	7052.2341	-0.0134
5	3	2	1^+	←	4	2	2	1^+	7113.7745	-0.0073
5	3	3	1^+	←	4	2	3	1^+	7145.4768	-0.0015
7	2	5	1^+	←	6	1	5	1^+	7364.9918	-0.0129
4	4	1	1^+	←	3	3	1	1^+	7529.7872	0.0103
6	3	3	1^+	←	5	2	3	1^+	7907.8748	-0.0134
6	3	4	1^+	←	5	2	4	1^+	7979.3200	0.0033
7	2	6	1^+	←	6	1	6	1^+	7998.2346	0.0032
5	1	4	1^+	←	4	2	2	1^+	2538.3668	-0.0023
					**$0^- \leftarrow 0^-$**					
4	0	4	0^-	←	3	1	2	0^-	2502.4752	-0.0063
3	1	2	0^-	←	2	0	2	0^-	3194.6489	0.0224
7	1	7	0^-	←	6	2	5	0^-	3257.0199	-0.0030
6	1	5	0^-	←	5	2	3	0^-	3432.7288	-0.0064
2	2	0	0^-	←	1	1	0	0^-	3440.3979	-0.0087
2	2	1	0^-	←	1	1	1	0^-	3481.7092	-0.0046
4	1	3	0^-	←	3	0	3	0^-	4104.2460	0.0111
3	2	1	0^-	←	2	1	1	0^-	4224.6285	-0.0056
7	1	6	0^-	←	6	2	4	0^-	4302.5453	0.0143
3	2	2	0^-	←	2	1	2	0^-	4343.7968	0.0069
8	0	8	0^-	←	7	1	6	0^-	4982.5952	0.0098
4	2	2	0^-	←	3	1	2	0^-	5000.2684	-0.0024
5	1	4	0^-	←	4	0	4	0^-	5042.7516	-0.0126
4	2	3	0^-	←	3	1	3	0^-	5227.0952	0.0096
9	0	9	0^-	←	8	1	7	0^-	5481.9470	-0.0230
3	3	0	0^-	←	2	2	0	0^-	5492.7762	-0.0133
3	3	1	0^-	←	2	2	1	0^-	5495.0268	-0.0133

J	K_a	K_c	v	←	J'	K_a'	K_c'	v′	ν_{obs}	ν_{obs}-ν_{calc}
5	2	3	0^-	←	4	1	3	0^-	5775.8202	-0.0091
9	1	8	0^-	←	8	2	6	0^-	5944.9161	0.0273
6	1	5	0^-	←	5	0	5	0^-	6014.5677	-0.0121
5	2	4	0^-	←	4	1	4	0^-	6131.5722	0.0041
4	3	1	0^-	←	3	2	1	0^-	6306.8322	0.0017
4	3	2	0^-	←	3	2	2	0^-	6317.8706	-0.0012
6	2	4	0^-	←	5	1	4	0^-	6560.5806	-0.0005
7	1	6	0^-	←	6	0	6	0^-	7023.3230	-0.0042
6	2	5	0^-	←	5	1	5	0^-	7057.1939	-0.0104
5	3	2	0^-	←	4	2	2	0^-	7112.8797	0.0081
5	3	3	0^-	←	4	2	3	0^-	7145.1093	-0.0009
7	2	5	0^-	←	6	1	5	0^-	7363.3910	0.0066
4	4	0	0^-	←	3	3	0	0^-	7527.5986	-0.0262
4	4	1	0^-	←	3	3	1	0^-	7527.6770	-0.0230
6	3	3	0^-	←	5	2	3	0^-	7906.6142	0.0096
6	3	4	0^-	←	5	2	4	0^-	7978.9912	-0.0026
					**$1^- \leftarrow 1^-$**					
2	1	1	1^-	←	1	0	1	1^-	2308.2053	0.0105
4	0	4	1^-	←	3	1	2	1^-	2502.8398	0.0039
3	1	2	1^-	←	2	0	2	1^-	3191.5951	0.0010
5	0	5	1^-	←	4	1	3	1^-	3195.7944	0.0101
7	1	7	1^-	←	6	2	5	1^-	3260.1559	0.0011
6	1	5	1^-	←	5	2	3	1^-	3429.2528	0.0093
2	2	0	1^-	←	1	1	0	1^-	3440.3979	-0.0087
2	2	1	1^-	←	1	1	1	1^-	3481.0363	0.0052
6	0	6	1^-	←	5	1	4	1^-	3843.0661	0.0062
4	1	3	1^-	←	3	0	3	1^-	4100.1952	0.0060
3	2	1	1^-	←	2	1	1	1^-	4224.4591	0.0090
7	1	6	1^-	←	6	2	4	1^-	4300.2447	-0.0094
3	2	2	1^-	←	2	1	2	1^-	4341.9285	-0.0072
8	0	8	1^-	←	7	1	6	1^-	4987.3776	-0.0098
4	2	2	1^-	←	3	1	2	1^-	5000.1896	0.0077
5	1	4	1^-	←	4	0	4	1^-	5037.9848	0.0071
4	2	3	1^-	←	3	1	3	1^-	5224.0718	-0.0125
9	0	9	1^-	←	8	1	7	1^-	5486.4785	0.0082
3	3	0	1^-	←	2	2	0	1^-	5492.4792	0.0130
3	3	1	1^-	←	2	2	1	1^-	5494.6938	0.0134
5	2	3	1^-	←	4	1	3	1^-	5775.9756	0.0041
9	1	8	1^-	←	8	2	6	1^-	5944.3767	-0.0012
6	1	5	1^-	←	5	0	5	1^-	6009.4360	0.0053
5	2	4	1^-	←	4	1	4	1^-	6127.5887	-0.0043
4	3	1	1^-	←	3	2	1	1^-	6306.1253	0.0032
4	3	2	1^-	←	3	2	2	1^-	6317.0144	0.0047
6	2	4	1^-	←	5	1	4	1^-	6561.0790	0.0045
7	1	6	1^-	←	6	0	6	1^-	7018.2593	-0.0094
6	2	5	1^-	←	5	1	5	1^-	7052.5419	-0.0068
5	3	2	1^-	←	4	2	2	1^-	7112.1313	-0.0051
5	3	3	1^-	←	4	2	3	1^-	7144.0183	0.0025
7	2	5	1^-	←	6	1	5	1^-	7364.3661	-0.0117
4	4	0	1^-	←	3	3	0	1^-	7527.2999	0.0108
4	4	1	1^-	←	3	3	1	1^-	7527.3778	0.0138
6	3	3	1^-	←	5	2	3	1^-	7906.1564	-0.0080
6	3	4	1^-	←	5	2	4	1^-	7977.9784	0.0047
7	2	6	1^-	←	6	1	6	1^-	7998.8943	0.0054
5	1	4	1^-	←	4	2	2	1^-	2540.6363	0.0046
					**^{c}*Q*-branch**					
					**$0^+ \leftarrow 0^+$**					
7	3	5	0^+	←	7	2	5	0^+	2849.5416	-0.0032
5	3	3	0^+	←	5	2	3	0^+	2981.4213	0.0146

J	K_a	K_c	v	←	J'	K_a'	K_c'	v′	ν_{obs}	ν_{obs}-ν_{calc}
6	3	3	0^+	←	6	2	5	0^+	3087.2331	0.0133
6	4	2	0^+	←	6	3	4	0^+	4247.4198	-0.0007
5	4	2	0^+	←	5	3	2	0^+	4247.8002	-0.0136
					**$1^+ \leftarrow 1^+$**					
7	3	5	1^+	←	7	2	5	1^+	2850.4100	0.0013
5	3	3	1^+	←	5	2	3	1^+	2983.0785	-0.0003
4	3	1	1^+	←	4	2	3	1^+	3049.4227	0.0005
5	3	2	1^+	←	5	2	4	1^+	3063.0127	-0.0072
6	3	3	1^+	←	6	2	5	1^+	3087.9772	-0.0032
8	4	5	1^+	←	8	3	5	1^+	4207.4517	0.0002
7	4	4	1^+	←	7	3	4	1^+	4229.2066	-0.0089
7	4	3	1^+	←	7	3	5	1^+	4245.3827	-0.0025
5	4	2	1^+	←	5	3	2	1^+	4249.6537	0.0125
5	4	1	1^+	←	5	3	3	1^+	4251.8050	0.0102
6	5	2	1^+	←	6	4	2	1^+	5467.2467	0.0119
6	5	2	1^+	←	6	4	3	1^+	5467.3316	0.0020
7	6	2	1^+	←	7	5	2	1^+	6683.2811	-0.0096
7	6	1	1^+	←	7	5	3	1^+	6683.2811	-0.0096
					**$0^- \leftarrow 0^-$**					
7	3	5	0^-	←	7	2	5	0^-	2846.7984	-0.0042
6	3	4	0^-	←	6	2	4	0^-	2926.0440	0.0192
5	3	3	0^-	←	5	2	3	0^-	2979.3444	0.0124
4	3	1	0^-	←	4	2	3	0^-	3046.2530	-0.0180
5	3	2	0^-	←	5	2	4	0^-	3060.2834	-0.0011
6	3	3	0^-	←	6	2	5	0^-	3085.6457	0.0138
7	3	4	0^-	←	7	2	6	0^-	3127.6314	0.0169
8	4	5	0^-	←	8	3	5	0^-	4204.3345	0.0065
6	4	3	0^-	←	6	3	3	0^-	4238.3203	0.0053
6	4	2	0^-	←	6	3	4	0^-	4244.8317	0.0087
5	4	2	0^-	←	5	3	2	0^-	4245.2189	-0.0160
5	4	1	0^-	←	5	3	3	0^-	4247.4198	-0.0007
9	5	5	0^-	←	9	4	5	0^-	5445.6561	0.0110
9	5	4	0^-	←	9	4	6	0^-	5448.4099	-0.0006
8	5	4	0^-	←	8	4	4	0^-	5453.5472	0.0113
8	5	3	0^-	←	8	4	5	0^-	5454.6177	0.0134
7	5	3	0^-	←	7	4	3	0^-	5458.7109	0.0166
7	5	2	0^-	←	7	4	4	0^-	5459.0642	0.0142
6	5	1	0^-	←	6	4	2	0^-	5461.9059	-0.0124
6	5	2	0^-	←	6	4	3	0^-	5462.0042	-0.0098
8	6	3	0^-	←	8	5	3	0^-	6674.8118	0.0060
8	6	2	0^-	←	8	5	4	0^-	6674.8118	0.0060
7	6	2	0^-	←	7	5	2	0^-	6677.0970	-0.0263
					**$1^- \leftarrow 1^-$**					
7	3	5	1^-	←	7	2	5	1^-	2847.5851	0.0049
6	3	4	1^-	←	6	2	4	1^-	2927.2432	-0.0029
5	3	3	1^-	←	5	2	3	1^-	2980.7681	-0.0015
4	3	1	1^-	←	4	2	3	1^-	3047.4673	0.0100
5	3	2	1^-	←	5	2	4	1^-	3061.1517	-0.0059
6	3	3	1^-	←	6	2	5	1^-	3086.2760	0.0015
7	3	4	1^-	←	7	2	6	1^-	3128.2146	0.0074
8	4	5	1^-	←	8	3	5	1^-	4204.4712	-0.0044
7	4	4	1^-	←	7	3	4	1^-	4226.3363	-0.0026
6	4	3	1^-	←	6	3	3	1^-	4239.3988	-0.0033
6	4	2	1^-	←	6	3	4	1^-	4245.9163	-0.0091
5	4	2	1^-	←	5	3	2	1^-	4246.8092	0.0116
5	4	1	1^-	←	5	3	3	1^-	4248.9813	0.0129
9	5	5	1^-	←	9	4	5	1^-	5445.6561	0.0110
9	5	4	1^-	←	9	4	6	1^-	5448.4099	- 0.0006
8	5	4	1^-	←	8	4	4	1^-	5453.9638	-0.0161

J	K_a	K_c	v	←	J'	K_a'	K_c'	v′	ν_{obs}	ν_{obs}-ν_{calc}
8	5	3	1^-	←	8	4	5	1^-	5455.0323	-0.0089
7	5	3	1^-	←	7	4	3	1^-	5459.7187	-0.0110
7	5	2	1^-	←	7	4	4	1^-	5460.0725	-0.0112
6	5	1	1^-	←	6	4	3	1^-	5463.7290	0.0064
7	6	1	1^-	←	7	5	3	1^-	6678.9211	0.0086

Table S11 Measured frequencies (ν_{obs}) in MHz and residuals ($\nu_{obs} - \nu_{calc}$) in MHz of the rotational transitions of the parent species of Phe-D_2O.

					***a* R-branch**					
J	K_a	K_c	v	←	J'	K_a'	K_c'	v′	ν_{obs}	ν_{obs}-ν_{calc}
					**$0^+ \leftarrow 1^+$**					
3	1	3	0^+	←	2	1	2	1^+	2389.0976	-0.0147
3	0	3	0^+	←	2	0	2	1^+	2449.4295	-0.0034
3	1	2	0^+	←	2	1	1	1^+	2526.1023	-0.0107
4	1	4	0^+	←	3	1	3	1^+	3184.9042	-0.0069
4	0	4	0^+	←	3	0	3	1^+	3256.8263	0.0049
4	2	3	0^+	←	3	2	2	1^+	3278.9222	0.0148
4	1	3	0^+	←	3	1	2	1^+	3367.7024	0.0216
5	1	5	0^+	←	4	1	4	1^+	3978.6654	0.0035
5	0	5	0^+	←	4	0	4	1^+	4055.4511	0.0048
5	2	4	0^+	←	4	2	3	1^+	4097.6637	0.0078
5	2	3	0^+	←	4	2	2	1^+	4145.3611	0.0110
5	1	4	0^+	←	4	1	3	1^+	4206.2077	0.0074
6	1	6	0^+	←	5	1	5	1^+	4770.0486	-0.0050
6	0	6	0^+	←	5	0	5	1^+	4844.8315	0.0001
6	2	5	0^+	←	5	2	4	1^+	4914.2364	0.0063
6	3	4	0^+	←	5	3	3	1^+	4936.0373	0.0047
6	3	3	0^+	←	5	3	2	1^+	4940.7142	0.0007
6	2	4	0^+	←	5	2	3	1^+	4993.9609	-0.0136
6	1	5	0^+	←	5	1	4	1^+	5040.3671	-0.0191
7	1	7	0^+	←	6	1	6	1^+	5558.9546	0.0108
7	0	7	0^+	←	6	0	6	1^+	5626.0638	-0.0043
7	2	6	0^+	←	6	2	5	1^+	5728.0723	-0.0158
7	3	5	0^+	←	6	3	4	1^+	5762.2654	-0.0087
7	3	4	0^+	←	6	3	3	1^+	5772.6644	0.0113
7	2	5	0^+	←	6	2	4	1^+	5847.4185	-0.0115
7	1	6	0^+	←	6	1	5	1^+	5868.7806	-0.0099
8	1	8	0^+	←	7	1	7	1^+	6345.3541	-0.0069
8	0	8	0^+	←	7	0	7	1^+	6401.4650	0.0057
8	3	5	0^+	←	7	3	4	1^+	6608.4647	0.0016
8	1	7	0^+	←	7	1	6	1^+	6689.9491	-0.0005
8	2	6	0^+	←	7	2	5	1^+	6702.7417	0.0001
9	1	9	0^+	←	8	1	8	1^+	7129.4777	-0.0016
9	0	9	0^+	←	8	0	8	1^+	7173.6269	0.0010
9	2	8	0^+	←	8	2	7	1^+	7345.9998	-0.0118
9	1	8	0^+	←	8	1	7	1^+	7502.5364	0.0266
9	2	7	0^+	←	8	2	6	1^+	7556.7787	0.0059
10	1	10	0^+	←	9	1	9	1^+	7911.5728	-0.0008
					**$1^+ \leftarrow 0^+$**					
3	0	3	1^+	←	2	0	2	0^+	2455.7116	0.0008
4	1	4	1^+	←	3	1	3	0^+	3191.8542	0.0126
4	0	4	1^+	←	3	0	3	0^+	3261.0452	-0.0025
4	2	3	1^+	←	3	2	2	0^+	3283.5705	0.0082
4	1	3	1^+	←	3	1	2	0^+	3368.5087	-0.0033
5	1	5	1^+	←	4	1	4	0^+	3984.0874	0.0002
5	0	5	1^+	←	4	0	4	0^+	4057.9251	-0.0033
5	2	4	1^+	←	4	2	3	0^+	4099.2032	-0.0126

J	K_a	K_c	v	←	J'	K_a'	K_c'	v′	ν_{obs}	ν_{obs}-ν_{calc}
5	3	3	1^+	←	4	3	2	0^+	4113.0797	0.0088
5	3	2	1^+	←	4	3	1	0^+	4114.8319	-0.0058
5	2	3	1^+	←	4	2	2	0^+	4146.0774	0.0049
5	1	4	1^+	←	4	1	3	0^+	4203.5264	-0.0075
6	1	6	1^+	←	5	1	5	0^+	4774.0707	0.0004
6	0	6	1^+	←	5	0	5	0^+	4846.1155	0.0040
6	2	5	1^+	←	5	2	4	0^+	4912.9375	-0.0089
6	3	4	1^+	←	5	3	3	0^+	4935.8576	-0.0081
6	3	3	1^+	←	5	3	2	0^+	4940.5427	-0.0068
6	2	4	1^+	←	5	2	3	0^+	4991.5908	0.0133
6	1	5	1^+	←	5	1	4	0^+	5034.9854	0.0068
7	1	7	1^+	←	6	1	6	0^+	5561.7631	0.0045
7	0	7	1^+	←	6	0	6	0^+	5626.6933	-0.0042
7	2	6	1^+	←	6	2	5	0^+	5724.5204	-0.0072
7	3	5	1^+	←	6	3	4	0^+	5759.5356	0.0082
7	3	4	1^+	←	6	3	3	0^+	5769.9466	-0.0072
7	2	5	1^+	←	6	2	4	0^+	5842.8458	0.0047
7	1	6	1^+	←	6	1	5	0^+	5861.8220	0.0226
8	1	8	1^+	←	7	1	7	0^+	6347.2288	-0.0045
8	0	8	1^+	←	7	0	7	0^+	6401.8374	0.0009
8	2	6	1^+	←	7	2	5	0^+	6697.3683	-0.0040
9	1	9	1^+	←	8	1	8	0^+	7130.6670	-0.0013
9	0	9	1^+	←	8	0	8	0^+	7173.9210	-0.0148
9	2	8	1^+	←	8	2	7	0^+	7340.2251	0.0180
9	1	8	1^+	←	8	1	7	0^+	7495.8369	-0.0084
9	2	7	1^+	←	8	2	6	0^+	7552.0831	-0.0037
10	1	10	1^+	←	9	1	9	0^+	7912.3058	0.0066
					**$0^- \leftarrow 1^-$**					
3	1	3	0^-	←	2	1	2	1^-	2389.0976	-0.0147
3	0	3	0^-	←	2	0	2	1^-	2449.4295	0.0034
3	1	2	0^-	←	2	1	1	1^-	2526.1023	-0.0107
4	1	4	0^-	←	3	1	3	1^-	3184.9671	0.0006
4	0	4	0^-	←	3	0	3	1^-	3256.8970	0.0105
4	2	3	0^-	←	3	2	2	1^-	3278.9222	0.0148
4	1	3	0^-	←	3	1	2	1^-	3367.7024	0.0216
5	1	5	0^-	←	4	1	4	1^-	3978.7929	0.0052
5	0	5	0^-	←	4	0	4	1^-	4055.5954	-0.0010
5	2	4	0^-	←	4	2	3	1^-	4097.7613	0.0124
5	3	3	0^-	←	4	3	2	1^-	4109.9931	0.0011
5	3	2	0^-	←	4	3	1	1^-	4111.7408	-0.0185
5	2	3	0^-	←	4	2	2	1^-	4145.3611	0.0110
5	1	4	0^-	←	4	1	3	1^-	4206.3021	0.0102
6	1	6	0^-	←	5	1	5	1^-	4770.2499	-0.0003
6	0	6	0^-	←	5	0	5	1^-	4845.0604	-0.0055
6	2	5	0^-	←	5	2	4	1^-	4914.4069	0.0083
6	3	4	0^-	←	5	3	3	1^-	4936.1523	0.0062
6	3	3	0^-	←	5	3	2	1^-	4940.8251	0.0089
6	2	4	0^-	←	5	2	3	1^-	4994.0343	-0.0081
6	1	5	0^-	←	5	1	4	1^-	5040.5325	-0.0111
7	1	7	0^-	←	6	1	6	1^-	5559.2098	0.0006
7	0	7	0^-	←	6	0	6	1^-	5626.3696	-0.0109
7	2	6	0^-	←	6	2	5	1^-	5728.3149	-0.0097
7	3	5	0^-	←	6	3	4	1^-	5762.4420	-0.0034
7	3	4	0^-	←	6	3	3	1^-	5772.8050	0.0046
7	2	5	0^-	←	6	2	4	1^-	5847.5058	-0.0123
7	1	6	0^-	←	6	1	5	1^-	5868.9994	-0.0037
8	1	8	0^-	←	7	1	7	1^-	6345.6805	-0.0111
8	0	8	0^-	←	7	0	7	1^-	6401.8374	0.0009
8	3	5	0^-	←	7	3	4	1^-	6608.6287	-0.0006

J	K_a	K_c	v	←	J'	K_a'	K_c'	v′	ν_{obs}	ν_{obs}-ν_{calc}
8	1	7	0^-	←	7	1	6	1^-	6690.2100	0.0011
8	2	6	0^-	←	7	2	5	1^-	6702.8358	0.0004
9	1	9	0^-	←	8	1	8	1^-	7129.8691	-0.0018
9	0	9	0^-	←	8	0	8	1^-	7174.0665	0.0012
9	2	8	0^-	←	8	2	7	1^-	7346.3385	-0.0153
9	1	8	0^-	←	8	1	7	1^-	7502.8377	0.0239
9	2	7	0^-	←	8	2	6	1^-	7556.8675	0.0032
10	1	10	0^-	←	9	1	9	1^-	7912.0319	0.0099
					**$1^- \leftarrow 0^-$**					
3	1	3	1^-	←	2	1	2	0^-	2397.7587	0.0203
3	0	3	1^-	←	2	0	2	0^-	2455.8832	0.0031
3	1	2	1^-	←	2	1	1	0^-	2530.7031	-0.0049
4	1	4	1^-	←	3	1	3	0^-	3192.1116	0.0066
4	0	4	1^-	←	3	0	3	0^-	3261.2265	-0.0015
4	2	3	1^-	←	3	2	2	0^-	3283.7286	0.0111
4	1	3	1^-	←	3	1	2	0^-	3368.5087	-0.0033
5	1	5	1^-	←	4	1	4	0^-	3984.3642	-0.0069
5	0	5	1^-	←	4	0	4	0^-	4058.1382	0.0002
5	2	4	1^-	←	4	2	3	0^-	4099.3374	-0.0092
5	3	3	1^-	←	4	3	2	0^-	4113.2107	-0.0038
5	3	2	1^-	←	4	3	1	0^-	4114.9721	-0.0052
5	2	3	1^-	←	4	2	2	0^-	4146.1318	0.0115
5	1	4	1^-	←	4	1	3	0^-	4203.5264	-0.0075
6	1	6	1^-	←	5	1	5	0^-	4774.3739	-0.0059
6	0	6	1^-	←	5	0	5	0^-	4846.3721	0.0026
6	2	5	1^-	←	5	2	4	0^-	4913.0603	-0.0022
6	3	4	1^-	←	5	3	3	0^-	4935.9670	-0.0070
6	3	3	1^-	←	5	3	2	0^-	4940.6364	-0.0111
6	2	4	1^-	←	5	2	3	0^-	4991.5908	0.0133
6	1	5	1^-	←	5	1	4	0^-	5034.9854	0.0068
7	1	7	1^-	←	6	1	6	0^-	5562.1010	0.0013
7	0	7	1^-	←	6	0	6	0^-	5627.0116	-0.0054
7	2	6	1^-	←	6	2	5	0^-	5724.6467	0.0023
7	3	5	1^-	←	6	3	4	0^-	5759.6212	0.0056
7	3	4	1^-	←	6	3	3	0^-	5770.0188	-0.0018
7	2	5	1^-	←	6	2	4	0^-	5842.8458	0.0047
7	1	6	1^-	←	6	1	5	0^-	5861.8220	0.0226
8	1	8	1^-	←	7	1	7	0^-	6347.6071	-0.0052
8	0	8	1^-	←	7	0	7	0^-	6402.2104	-0.0068
8	3	5	1^-	←	7	3	4	0^-	6604.3608	-0.0003
8	1	7	1^-	←	7	1	6	0^-	6682.6660	0.0070
8	2	6	1^-	←	7	2	5	0^-	6697.3683	-0.0040
9	1	9	1^-	←	8	1	8	0^-	7131.0936	0.0033
9	0	9	1^-	←	8	0	8	0^-	7174.3699	-0.0117
9	2	8	1^-	←	8	2	7	0^-	7340.3924	0.0136
9	1	8	1^-	←	8	1	7	0^-	7495.9521	-0.0211
9	2	7	1^-	←	8	2	6	0^-	7552.0831	-0.0037
10	1	10	1^-	←	9	1	9	0^-	7912.7791	0.0119
					**^cR-branch**					
					**$0^+ \leftarrow 0^+$**					
2	1	1	0^+	←	1	0	1	0^+	2326.0748	0.0055
3	1	2	0^+	←	2	0	2	0^+	3215.8014	0.0253
2	2	0	0^+	←	1	1	0	0^+	3470.0719	-0.0040
2	2	1	0^+	←	1	1	1	0^+	3512.9264	0.0019
4	1	3	0^+	←	3	0	3	0^+	4131.7409	0.0236
3	2	1	0^+	←	2	1	1	0^+	4255.6943	-0.0091
4	2	2	0^+	←	3	1	2	0^+	5032.7545	-0.0237
4	2	3	0^+	←	3	1	3	0^+	5267.5733	0.0033
3	3	0	0^+	←	2	2	0	0^+	5542.5001	-0.0085

J	K_a	K_c	v	←	*J*′	K_a′	K_c′	v′	ν_{obs}	ν_{obs}-ν_{calc}
3	3	1	0^+	←	2	2	1	0^+	5544.9003	-0.0077
5	2	3	0^+	←	4	1	3	0^+	5810.3348	-0.0047
6	1	5	0^+	←	5	0	5	0^+	6059.1203	-0.0065
5	2	4	0^+	←	4	1	4	0^+	6178.0264	0.0110
4	3	1	0^+	←	3	2	1	0^+	6358.8058	0.0141
4	3	2	0^+	←	3	2	2	0^+	6370.5386	-0.0045
6	2	4	0^+	←	5	1	4	0^+	6598.1199	-0.0056
7	1	6	0^+	←	6	0	6	0^+	7078.9580	-0.0079
6	2	5	0^+	←	5	1	5	0^+	7110.5126	0.0002
5	3	2	0^+	←	4	2	2	0^+	7166.6305	0.0232
5	3	3	0^+	←	4	2	3	0^+	7200.9214	-0.0022
7	2	5	0^+	←	6	1	5	0^+	7405.4133	-0.0005
4	4	0	0^+	←	3	3	0	0^+	7596.8180	-0.0051
4	4	1	0^+	←	3	3	1	0^+	7596.9032	-0.0024
6	3	3	0^+	←	5	2	3	0^+	7961.4059	0.0097
					**$1^+ \leftarrow 1^+$**					
3	1	2	1^+	←	2	0	2	1^+	3213.4742	0.0117
2	2	0	1^+	←	1	1	0	1^+	3470.0719	-0.0040
2	2	1	1^+	←	1	1	1	1^+	3512.4091	0.0070
4	1	3	1^+	←	3	0	3	1^+	4128.5677	0.0028
3	2	1	1^+	←	2	1	1	1^+	4255.5533	0.0104
4	2	2	1^+	←	3	1	2	1^+	5032.6576	-0.0070
5	1	4	1^+	←	4	0	4	1^+	5074.1953	0.0083
4	2	3	1^+	←	3	1	3	1^+	5265.2561	-0.0144
3	3	0	1^+	←	2	2	0	1^+	5542.2724	0.0093
3	3	1	1^+	←	2	2	1	1^+	5544.6425	0.0100
5	2	3	1^+	←	4	1	3	1^+	5810.3348	-0.0047
6	1	5	1^+	←	5	0	5	1^+	6055.0125	0.0056
5	2	4	1^+	←	4	1	4	1^+	6174.9382	-0.0059
4	3	1	1^+	←	3	2	1	1^+	6358.2317	-0.0027
4	3	2	1^+	←	3	2	2	1^+	6369.8746	0.0003
6	2	4	1^+	←	5	1	4	1^+	6598.3820	0.0119
7	1	6	1^+	←	6	0	6	1^+	7074.8114	-0.0069
6	2	5	1^+	←	5	1	5	1^+	7106.8689	-0.0056
5	3	2	1^+	←	4	2	2	1^+	7166.0115	-0.0055
5	3	3	1^+	←	4	2	3	1^+	7200.0509	-0.0008
7	2	5	1^+	←	6	1	5	1^+	7406.0042	0.0065
4	4	0	1^+	←	3	3	0	1^+	7596.5876	-0.0001
4	4	1	1^+	←	3	3	1	1^+	7596.6725	0.0026
6	3	3	1^+	←	5	2	3	1^+	7961.0572	-0.0010
					**$0^- \leftarrow 0^-$**					
2	1	1	0^-	←	1	0	1	0^-	2326.1786	0.0018
3	1	2	0^-	←	2	0	2	0^-	3215.8879	0.0229
2	2	0	0^-	←	1	1	0	0^-	3470.4492	-0.0021
2	2	1	0^-	←	1	1	1	0^-	3513.2395	-0.0066
4	1	3	0^-	←	3	0	3	0^-	4131.7409	0.0236
3	2	1	0^-	←	2	1	1	0^-	4256.0790	-0.0115
3	2	2	0^-	←	2	1	2	0^-	4379.5099	-0.0136
4	2	2	0^-	←	3	1	2	0^-	5033.1917	-0.0076
5	1	4	0^-	←	4	0	4	0^-	5077.9093	-0.0053
4	2	3	0^-	←	3	1	3	0^-	5267.8731	-0.0018
3	3	0	0^-	←	2	2	0	0^-	5543.0596	-0.0016
3	3	1	0^-	←	2	2	1	0^-	5545.4544	-0.0027
6	1	5	0^-	←	5	0	5	0^-	6058.9798	-0.0071
5	2	4	0^-	←	4	1	4	0^-	6178.2855	0.0004
4	3	1	0^-	←	3	2	1	0^-	6359.3656	0.0040
4	3	2	0^-	←	3	2	2	0^-	6371.1133	0.0000
6	2	4	0^-	←	5	1	4	0^-	6598.5395	-0.0062
7	1	6	0^-	←	6	0	6	0^-	7078.6737	-0.0060

J	K_a	K_c	v	←	J'	K_a'	K_c'	v′	ν_{obs}	ν_{obs}-ν_{calc}
6	2	5	0^-	←	5	1	5	0^-	7110.7194	-0.0082
5	3	2	0^-	←	4	2	2	0^-	7167.2249	0.0132
5	3	3	0^-	←	4	2	3	0^-	7201.5040	-0.0010
7	2	5	0^-	←	6	1	5	0^-	7405.7762	-0.0068
4	4	0	0^-	←	3	3	0	0^-	7597.5877	-0.0050
4	4	1	0^-	←	3	3	1	0^-	7597.6743	-0.0007
6	3	3	0^-	←	5	2	3	0^-	7962.1002	0.0163
					$1^- \leftarrow 1^-$					
3	1	2	1^-	←	2	0	2	1^-	3213.4742	0.0117
2	2	0	1^-	←	1	1	0	1^-	3470.3911	-0.0031
2	2	1	1^-	←	1	1	1	1^-	3512.7224	0.0117
4	1	3	1^-	←	3	0	3	1^-	4128.5033	-0.0058
3	2	1	1^-	←	2	1	1	1^-	4255.9292	-0.0004
3	2	2	1^-	←	2	1	2	1^-	4378.0422	-0.0243
4	2	2	1^-	←	3	1	2	1^-	5033.0969	0.0090
5	1	4	1^-	←	4	0	4	1^-	5074.0542	0.0144
4	2	3	1^-	←	3	1	3	1^-	5265.4860	-0.0166
3	3	0	1^-	←	2	2	0	1^-	5542.8237	0.0102
3	3	1	1^-	←	2	2	1	1^-	5545.1871	0.0086
5	2	3	1^-	←	4	1	3	1^-	5810.8090	0.0138
6	1	5	1^-	←	5	0	5	1^-	6054.7524	0.0099
5	2	4	1^-	←	4	1	4	1^-	6175.1044	-0.0123
4	3	1	1^-	←	3	2	1	1^-	6358.8058	0.0141
4	3	2	1^-	←	3	2	2	1^-	6370.4292	0.0012
6	2	4	1^-	←	5	1	4	1^-	6598.8166	0.0081
7	1	6	1^-	←	6	0	6	1^-	7074.4247	0.0112
6	2	5	1^-	←	5	1	5	1^-	7106.9625	-0.0139
5	3	2	1^-	←	4	2	2	1^-	7166.6305	0.0232
5	3	3	1^-	←	4	2	3	1^-	7200.6092	-0.0012
7	2	5	1^-	←	6	1	5	1^-	7406.3887	-0.0099
4	4	0	1^-	←	3	3	0	1^-	7597.3514	-0.0032
4	4	1	1^-	←	3	3	1	1^-	7597.4355	-0.0010
6	3	3	1^-	←	5	2	3	1^-	7961.7375	-0.0014

Table S12 Measured frequencies (ν_{obs}) in MHz and residuals ($\nu_{obs} - \nu_{calc}$) in MHz of the rotational transitions of the parent species of Phe-HDO.

					***a* R-branch**					
J	K_a	K_c	v	←	J'	K_a'	K_c'	v′	ν_{obs}	ν_{obs}-ν_{calc}
					$0^+ \leftarrow 1^-$					
3	1	3	0^+	←	2	1	2	1^-	2397.1446	-0.0026
3	0	3	0^+	←	2	0	2	1^-	2459.7312	0.0150
3	1	2	0^+	←	2	1	1	1^-	2540.4869	-0.0054
4	1	4	0^+	←	3	1	3	1^-	3193.3373	0.0016
4	0	4	0^+	←	3	0	3	1^-	3267.4857	-0.0017
4	2	3	0^+	←	3	2	2	1^-	3291.9789	0.0043
4	2	2	0^+	←	3	2	1	1^-	3318.6245	-0.0083
4	1	3	0^+	←	3	1	2	1^-	3384.0393	0.0066
5	1	5	0^+	←	4	1	4	1^-	3987.2665	-0.0045
5	0	5	0^+	←	4	0	4	1^-	4065.8703	0.0044
5	2	4	0^+	←	4	2	3	1^-	4111.6622	0.0046
5	3	3	0^+	←	4	3	2	1^-	4125.9813	0.0005
5	3	2	0^+	←	4	3	1	1^-	4127.9993	0.0001
5	2	3	0^+	←	4	2	2	1^-	4163.5460	0.0027
5	1	4	0^+	←	4	1	3	1^-	4224.3597	0.0071
6	1	6	0^+	←	5	1	5	1^-	4778.6876	0.0030
6	0	6	0^+	←	5	0	5	1^-	4854.5989	0.0063

J	K_a	K_c	v	←	J'	K_a'	K_c'	v′	ν_{obs}	ν_{obs}-ν_{calc}
6	4	3	0^+	←	5	4	2	1^-	4950.2821	-0.0149
6	4	2	0^+	←	5	4	1	1^-	4950.3968	-0.0064
6	3	4	0^+	←	5	3	3	1^-	4953.5794	-0.0059
6	3	3	0^+	←	5	3	2	1^-	4958.9186	-0.0053
6	2	4	0^+	←	5	2	3	1^-	5015.7193	0.0000
6	1	5	0^+	←	5	1	4	1^-	5060.2667	0.0015
7	1	7	0^+	←	6	1	6	1^-	5567.5095	0.0052
7	0	7	0^+	←	6	0	6	1^-	5635.0544	0.0049
7	2	6	0^+	←	6	2	5	1^-	5743.8337	0.0004
7	5	3	0^+	←	6	5	2	1^-	5774.1780	-0.0086
7	5	2	0^+	←	6	5	1	1^-	5774.1780	-0.0086
7	4	4	0^+	←	6	4	3	1^-	5778.0488	-0.0045
7	4	3	0^+	←	6	4	2	1^-	5778.3929	-0.0007
7	3	5	0^+	←	6	3	4	1^-	5781.6935	0.0025
7	3	4	0^+	←	6	3	3	1^-	5793.5386	0.0006
7	2	5	0^+	←	6	2	4	1^-	5873.3398	-0.0046
7	1	6	0^+	←	6	1	5	1^-	5890.3726	0.0042
8	1	8	0^+	←	7	1	7	v	6353.8166	0.0075
8	0	8	0^+	←	7	0	7	1^-	6409.7285	0.0072
8	2	7	0^+	←	7	2	6	1^-	6555.5178	-0.0043
8	6	3	0^+	←	7	6	2	1^-	6598.1120	0.0042
8	6	2	0^+	←	7	6	1	1^-	6598.1120	0.0042
8	4	5	0^+	←	7	4	4	1^-	6606.8904	0.0016
8	4	4	0^+	←	7	4	3	1^-	6607.8247	0.0062
8	3	6	0^+	←	7	3	5	1^-	6609.8679	-0.0067
8	3	5	0^+	←	7	3	4	1^-	6633.0568	-0.0018
8	1	7	0^+	←	7	1	6	1^-	6713.0774	-0.0055
8	2	6	0^+	←	7	2	5	1^-	6733.2583	-0.0068
9	1	9	0^+	←	8	1	8	1^-	7137.8073	-0.0006
9	0	9	0^+	←	8	0	8	1^-	7181.3597	-0.0100
9	2	8	0^+	←	8	2	7	1^-	7363.8179	-0.0121
9	6	4	0^+	←	8	6	3	1^-	7424.8514	-0.0084
9	6	3	0^+	←	8	6	2	1^-	7424.8514	-0.0084
9	5	5	0^+	←	8	5	4	1^-	7429.4089	0.0130
9	5	4	0^+	←	8	5	3	1^-	7429.4089	0.0130
9	4	6	0^+	←	8	4	5	1^-	7436.8243	-0.0044
9	3	7	0^+	←	8	3	6	1^-	7437.5666	-0.0025
9	4	5	0^+	←	8	4	4	1^-	7439.0534	0.0142
9	3	6	0^+	←	8	3	5	1^-	7478.7193	0.0143
9	1	8	0^+	←	8	1	7	1^-	7526.8048	0.0000
9	2	7	0^+	←	8	2	6	1^-	7592.0829	0.0193
10	1	10	0^+	←	9	1	9	1^-	7919.8208	-0.0041
10	0	10	0^+	←	9	0	9	1^-	7952.1484	-0.0009
					**$1^- \leftarrow 0^+$**					
3	1	3	1^-	←	2	1	2	0^-	2397.5765	0.0061
3	0	3	1^-	←	2	0	2	0^-	2460.0542	0.0068
3	1	2	1^-	←	2	1	1	0^-	2540.7219	-0.0041
4	1	4	1^-	←	3	1	3	0^-	3193.6988	0.0002
4	0	4	1^-	←	3	0	3	0^-	3267.7119	-0.0025
4	2	3	1^-	←	3	2	2	0^-	3292.2198	0.0017
4	2	2	1^-	←	3	2	1	0^-	3318.8457	-0.0053
4	1	3	1^-	←	3	1	2	0^-	3384.0846	-0.0139
5	1	5	1^-	←	4	1	4	0^-	3987.5511	0.0005
5	0	5	1^-	←	4	0	4	0^-	4066.0074	0.0003
5	2	4	1^-	←	4	2	3	0^-	4111.7477	-0.0106
5	3	3	1^-	←	4	3	2	0^-	4126.1547	0.0040
5	3	2	1^-	←	4	3	1	0^-	4128.1678	-0.0010
5	2	3	1^-	←	4	2	2	0^-	4163.5460	0.0027
5	1	4	1^-	←	4	1	3	0^-	4224.2362	-0.0110

J	K_a	K_c	v	←	J'	K_a'	K_c'	v′	ν_{obs}	ν_{obs}-ν_{calc}
6	1	6	1^-	←	5	1	5	0^+	4778.9014	0.0009
6	0	6	1^-	←	5	0	5	0^+	4854.6774	0.0026
6	2	5	1^-	←	5	2	4	0^+	4929.0490	0.0040
6	4	3	1^-	←	5	4	2	0^+	4950.3968	-0.0064
6	4	2	1^-	←	5	4	1	0^+	4950.4966	-0.0159
6	3	4	1^-	←	5	3	3	0^+	4953.5794	-0.0059
6	3	3	1^-	←	5	3	2	0^+	4958.9186	-0.0053
6	2	4	1^-	←	5	2	3	0^+	5015.6017	-0.0029
6	1	5	1^-	←	5	1	4	0^+	5060.0042	-0.0078
7	1	7	1^-	←	6	1	6	0^+	5567.6622	-0.0012
7	0	7	1^-	←	6	0	6	0^+	5635.0544	0.0049
7	2	6	1^-	←	6	2	5	0^+	5743.6652	-0.0059
7	5	3	1^-	←	6	5	2	0^+	5774.1780	-0.0086
7	5	2	1^-	←	6	5	1	0^+	5774.1780	-0.0086
7	4	4	1^-	←	6	4	3	0^+	5778.0031	0.0012
7	4	3	1^-	←	6	4	2	0^+	5778.3523	0.0097
7	3	5	1^-	←	6	3	4	0^+	5781.5614	0.0018
7	3	4	1^-	←	6	3	3	0^+	5793.4084	-0.0029
7	2	5	1^-	←	6	2	4	0^+	5873.1083	0.0058
7	1	6	1^-	←	6	1	5	0^+	5890.0269	0.0112
8	1	8	1^-	←	7	1	7	0^+	6353.9213	-0.0004
8	0	8	1^-	←	7	0	7	0^+	6409.7285	0.0072
8	2	7	1^-	←	7	2	6	0^+	6555.2643	-0.0039
8	6	3	1^-	←	7	6	2	0^+	6598.1120	0.0042
8	6	2	1^-	←	7	6	1	0^+	6598.1120	0.0042
8	4	5	1^-	←	7	4	4	0^+	6606.7218	0.0068
8	4	4	1^-	←	7	4	3	0^+	6607.6506	0.0017
8	3	6	1^-	←	7	3	5	0^+	6609.6536	0.0069
8	3	5	1^-	←	7	3	4	0^+	6632.8532	0.0089
8	1	7	1^-	←	7	1	6	0^+	6712.7061	0.0082
8	2	6	1^-	←	7	2	5	0^+	6732.9786	0.0056
9	1	9	1^-	←	8	1	8	0^+	7137.8822	-0.0040
9	0	9	1^-	←	8	0	8	0^+	7181.4200	0.0125
9	2	8	1^-	←	8	2	7	0^+	7363.5293	0.0040
9	6	4	1^-	←	8	6	3	0^+	7424.7250	0.0036
9	6	3	1^-	←	8	6	2	0^+	7424.7250	0.0036
9	5	5	1^-	←	8	5	4	0^+	7429.1904	-0.0092
9	5	4	1^-	←	8	5	3	0^+	7429.1904	-0.0092
9	3	7	1^-	←	8	3	6	0^+	7437.3191	0.0110
9	4	5	1^-	←	8	4	4	0^+	7438.8275	0.0060
9	3	6	1^-	←	8	3	5	0^+	7478.4965	-0.0069
9	1	8	1^-	←	8	1	7	0^+	7526.4525	-0.0111
9	2	7	1^-	←	8	2	6	0^+	7591.8291	-0.0063
10	1	10	1^-	←	9	1	9	0^+	7919.8208	-0.0041
10	0	10	1^-	←	9	0	9	0^+	7952.1964	0.0073
					**$0^- \leftarrow 1^+$**					
3	1	2	0^-	←	2	1	1	1^+	2540.4869	-0.0054
4	0	4	0^-	←	3	0	3	1^+	3267.4857	-0.0017
4	2	3	0^-	←	3	2	2	1^+	3291.9789	0.0043
4	2	2	0^-	←	3	2	1	1^+	3318.6245	-0.0083
4	1	3	0^-	←	3	1	2	1^+	3384.0393	0.0066
5	1	5	0^-	←	4	1	4	1^+	3987.2665	-0.0045
5	0	5	0^-	←	4	0	4	1^+	4065.8703	-0.0071
5	2	4	0^-	←	4	2	3	1^+	4111.6622	0.0046
5	3	3	0^-	←	4	3	2	1^+	4125.9813	0.0005
5	3	2	0^-	←	4	3	1	1^+	4127.9993	0.0001
5	2	3	0^-	←	4	2	2	1^+	4163.5460	0.0027
5	1	4	0^-	←	4	1	3	1^+	4224.3597	0.0071
6	1	6	0^-	←	5	1	5	1^+	4778.6876	0.0030

J	K_a	K_c	v	←	J'	K_a'	K_c'	v′	ν_{obs}	ν_{obs}-ν_{calc}
6	0	6	0^-	←	5	0	5	1^+	4854.5989	0.0063
6	4	3	0^-	←	5	4	2	1^+	4950.2821	-0.0149
6	4	2	0^-	←	5	4	1	1^+	4950.3968	-0.0064
6	3	4	0^-	←	5	3	3	1^+	4953.5794	-0.0059
6	3	3	0^-	←	5	3	2	1^+	4958.9186	-0.0053
6	2	4	0^-	←	5	2	3	1^+	5015.7193	0.0000
6	1	5	0^-	←	5	1	4	1^+	5060.2667	0.0015
7	1	7	0^-	←	6	1	6	1^+	5567.5095	0.0052
7	0	7	0^-	←	6	0	6	1^+	5635.0544	0.0049
7	2	6	0^-	←	6	2	5	1^+	5743.8337	0.0004
7	5	3	0^-	←	6	5	2	1^+	5774.1780	-0.0086
7	5	2	0^-	←	6	5	1	1^+	5774.1780	-0.0086
7	4	4	0^-	←	6	4	3	1^+	5778.0488	-0.0045
7	4	3	0^-	←	6	4	2	1^+	5778.3929	-0.0007
7	3	5	0^-	←	6	3	4	1^+	5781.6935	0.0025
7	3	4	0^-	←	6	3	3	1^+	5793.5386	0.0006
7	2	5	0^-	←	6	2	4	1^+	5873.3398	-0.0046
7	1	6	0^-	←	6	1	5	1^+	5890.3726	0.0042
8	1	8	0^-	←	7	1	7	1^+	6353.8166	0.0075
8	0	8	0^-	←	7	0	7	1^+	6409.7285	0.0072
8	2	7	0^-	←	7	2	6	1^+	6555.5178	-0.0043
8	6	3	0^-	←	7	6	2	1^+	6598.1120	0.0042
8	6	2	0^-	←	7	6	1	1^+	6598.1120	0.0042
8	4	5	0^-	←	7	4	4	1^+	6606.8904	0.0016
8	4	4	0^-	←	7	4	3	1^+	6607.8247	0.0062
8	3	6	0^-	←	7	3	5	1^+	6609.8679	-0.0067
8	3	5	0^-	←	7	3	4	1^+	6633.0568	-0.0018
8	1	7	0^-	←	7	1	6	1^+	6713.0774	-0.0055
8	2	6	0^-	←	7	2	5	1^+	6733.2583	-0.0068
9	1	9	0^-	←	8	1	8	1^+	7137.8073	-0.0006
9	0	9	0^-	←	8	0	8	1^+	7181.3589	-0.0180
9	2	8	0^-	←	8	2	7	1^+	7363.8179	-0.0121
9	6	4	0^-	←	8	6	3	1^+	7424.8514	-0.0084
9	6	3	0^-	←	8	6	2	1^+	7424.8514	-0.0084
9	5	5	0^-	←	8	5	4	1^+	7429.4089	0.0130
9	5	4	0^-	←	8	5	3	1^+	7429.4089	0.0130
9	4	6	0^-	←	8	4	5	1^+	7436.8243	-0.0044
9	3	7	0^-	←	8	3	6	1^+	7437.5666	-0.0025
9	4	5	0^-	←	8	4	4	1^+	7439.0534	0.0142
9	3	6	0^-	←	8	3	5	1^+	7478.7193	0.0143
9	1	8	0^-	←	8	1	7	1^+	7526.8048	0.0000
9	2	7	0^-	←	8	2	6	1^+	7592.0829	0.0193
10	1	10	0^-	←	9	1	9	1^+	7919.8208	-0.0041
10	0	10	0^-	←	9	0	9	1^+	7952.1484	-0.0009
					$1^+ \leftarrow 0^-$					
3	1	2	1^+	←	2	1	1	0^-	2540.7219	-0.0041
4	0	4	1^+	←	3	0	3	0^-	3267.7119	0.0025
4	2	3	1^+	←	3	2	2	0^-	3292.2198	0.0017
4	2	2	1^+	←	3	2	1	0^-	3318.8457	-0.0053
4	1	3	1^+	←	3	1	2	0^-	3384.0846	-0.0139
5	1	5	1^+	←	4	1	4	0^-	3987.5511	0.0005
5	0	5	1^+	←	4	0	4	0^-	4066.0074	0.0003
5	2	4	1^+	←	4	2	3	0^-	4111.7477	-0.0106
5	3	3	1^+	←	4	3	2	0^-	4126.1547	0.0040
5	3	2	1^+	←	4	3	1	0^-	4128.1678	-0.0010
5	2	3	1^+	←	4	2	2	0^-	4163.5460	0.0027
5	1	4	1^+	←	4	1	3	0^-	4224.2362	-0.0110
6	1	6	1^+	←	5	1	5	0^-	4778.9014	0.0009
6	0	6	1^+	←	5	0	5	0^-	4854.6774	0.0026

J	K_a	K_c	v	←	J'	K_a'	K_c'	v′	ν_{obs}	ν_{obs}-ν_{calc}
6	2	5	1^+	←	5	2	4	0^-	4929.0490	0.0040
6	4	3	1^+	←	5	4	2	0^-	4950.3970	-0.0120
6	4	2	1^+	←	5	4	1	0^-	4950.4966	-0.0159
6	3	4	1^+	←	5	3	3	0^-	4953.5794	-0.0059
6	3	3	1^+	←	5	3	2	0^-	4958.9186	-0.0053
6	2	4	1^+	←	5	2	3	0^-	5015.6017	-0.0029
6	1	5	1^+	←	5	1	4	0^-	5060.0042	-0.0078
7	1	7	1^+	←	6	1	6	0^-	5567.6622	-0.0012
7	0	7	1^+	←	6	0	6	0^-	5635.0544	0.0049
7	2	6	1^+	←	6	2	5	0^-	5743.6652	-0.0059
7	5	2	1^+	←	6	5	1	0^-	5774.1780	-0.0086
7	4	4	1^+	←	6	4	3	0^-	5778.0031	0.0012
7	4	3	1^+	←	6	4	2	0^-	5778.3523	0.0097
7	3	5	1^+	←	6	3	4	0^-	5781.5614	0.0018
7	3	4	1^+	←	6	3	3	0^-	5793.4084	-0.0029
7	2	5	1^+	←	6	2	4	0^-	5873.1083	0.0058
7	1	6	1^+	←	6	1	5	0^-	5890.0269	0.0112
8	1	8	1^+	←	7	1	7	0^-	6353.9213	-0.0004
8	0	8	1^+	←	7	0	7	0^-	6409.7285	0.0072
8	2	7	1^+	←	7	2	6	0^-	6555.2643	-0.0039
8	6	3	1^+	←	7	6	2	0^-	6598.1120	0.0042
8	6	2	1^+	←	7	6	1	0^-	6598.1120	0.0042
8	4	5	1^+	←	7	4	4	0^-	6606.7220	0.0020
8	4	4	1^+	←	7	4	3	0^-	6607.6506	0.0017
8	3	6	1^+	←	7	3	5	0^-	6609.6536	0.0069
8	3	5	1^+	←	7	3	4	0^-	6632.8530	-0.0038
8	1	7	1^+	←	7	1	6	0^-	6712.7061	0.0082
8	2	6	1^+	←	7	2	5	0^-	6732.9786	0.0056
9	1	9	1^+	←	8	1	8	0^-	7137.8822	-0.0040
9	0	9	1^+	←	8	0	8	0^-	7181.4200	0.0125
9	2	8	1^+	←	8	2	7	0^-	7363.5293	0.0040
9	6	3	1^+	←	8	6	2	0^-	7424.7250	0.0036
9	6	4	1^+	←	8	6	3	0^-	7424.7250	0.0036
9	5	5	1^+	←	8	5	4	0^-	7429.1904	-0.0092
9	5	4	1^+	←	8	5	3	0^-	7429.1904	-0.0092
9	4	6	1^+	←	8	4	5	0^-	7436.6092	-0.0016
9	3	7	1^+	←	8	3	6	0^-	7437.3191	0.0110
9	4	5	1^+	←	8	4	4	0^-	7438.8275	0.0060
9	3	6	1^+	←	8	3	5	0^-	7478.4965	-0.0069
9	1	8	1^+	←	8	1	7	0^-	7526.4525	-0.0111
9	2	7	1^+	←	8	2	6	0^-	7591.8291	-0.0063
10	1	10	1^+	←	9	1	9	0^-	7919.8208	-0.0041
10	0	10	1^+	←	9	0	9	0^-	7952.1964	0.0073
					**^{c}*R*-branch**					
					**$0^+ \leftarrow 0^+$**					
2	1	1	0^+	←	1	0	1	0^+	2348.7551	-0.0143
6	1	6	0^+	←	5	2	4	0^+	2521.1163	-0.0063
3	1	2	0^+	←	2	0	2	0^+	3244.9707	-0.0003
2	2	0	0^+	←	1	1	0	0^+	3515.7611	-0.0013
4	1	3	0^+	←	3	0	3	0^+	4169.1746	0.0088
7	1	6	0^+	←	6	2	4	0^+	4299.0808	0.0015
3	2	1	0^+	←	2	1	1	0^+	4302.3199	-0.0076
3	2	2	0^+	←	2	1	2	0^+	4432.1629	0.0106
8	0	8	0^+	←	7	1	6	0^+	4889.1534	0.0023
4	2	2	0^+	←	3	1	2	0^+	5080.4709	0.0106
5	1	4	0^+	←	4	0	4	0^+	5125.8766	-0.0006
4	2	3	0^+	←	3	1	3	0^+	5326.9338	0.0114
3	3	0	0^+	←	2	2	0	0^+	5619.3752	-0.0053
3	3	1	0^+	←	2	2	1	0^+	5621.9945	-0.0054

J	K_a	K_c	v	←	J'	K_a'	K_c'	v′	ν_{obs}	ν_{obs}-ν_{calc}
5	2	3	0^+	←	4	1	3	0^+	5859.9531	0.0150
6	1	5	0^+	←	5	0	5	0^+	6120.0810	-0.0012
5	2	4	0^+	←	4	1	4	0^+	6245.1263	-0.0008
4	3	1	0^+	←	3	2	1	0^+	6437.7903	-0.0150
4	3	2	0^+	←	3	2	2	0^+	6450.6605	-0.0053
6	2	4	0^+	←	5	1	4	0^+	6651.2984	-0.0017
7	1	6	0^+	←	6	0	6	0^+	7155.6359	0.0030
6	2	5	0^+	←	5	1	5	0^+	7186.7962	0.0055
5	3	2	0^+	←	4	2	2	0^+	7247.1378	-0.0221
5	3	3	0^+	←	4	2	3	0^+	7284.6206	-0.0145
7	2	5	0^+	←	6	1	5	0^+	7464.3509	-0.0162
4	4	0	0^+	←	3	3	0	0^+	7704.0989	0.0065
4	4	1	0^+	←	3	3	1	0^+	7704.1927	0.0064
					1^-← 1^-					
2	1	1	1^-	←	1	0	1	1^-	2348.6899	-0.0193
6	1	6	1^-	←	5	2	4	1^-	2521.1892	0.0016
3	1	2	1^-	←	2	0	2	1^-	3244.8503	-0.0191
2	2	0	1^-	←	1	1	0	1^-	3515.7611	-0.0013
4	1	3	1^-	←	3	0	3	1^-	4169.0176	-0.0047
7	1	6	1^-	←	6	2	4	1^-	4298.9844	0.0026
3	2	1	1^-	←	2	1	1	1^-	4302.3199	-0.0076
3	2	2	1^-	←	2	1	2	1^-	4432.0915	-0.0181
8	0	8	1^-	←	7	1	6	1^-	4889.3587	-0.0013
4	2	2	1^-	←	3	1	2	1^-	5080.4709	0.0106
5	1	4	1^-	←	4	0	4	1^-	5125.6867	-0.0090
4	2	3	1^-	←	3	1	3	1^-	5326.8241	0.0028
3	3	0	1^-	←	2	2	0	1^-	5619.3752	-0.0053
3	3	1	1^-	←	2	2	1	1^-	5621.9945	-0.0054
5	2	3	1^-	←	4	1	3	1^-	5859.9531	0.0150
6	1	5	1^-	←	5	0	5	1^-	6119.8740	0.0021
5	2	4	1^-	←	4	1	4	1^-	6244.9751	-0.0108
4	3	1	1^-	←	3	2	1	1^-	6437.7903	-0.0150
4	3	2	1^-	←	3	2	2	1^-	6450.6605	-0.0053
6	2	4	1^-	←	5	1	4	1^-	6651.2984	-0.0017
7	1	6	1^-	←	6	0	6	1^-	7155.4218	0.0116
6	2	5	1^-	←	5	1	5	1^-	7186.6131	-0.0021
5	3	2	1^-	←	4	2	2	1^-	7247.1378	-0.0221
5	3	3	1^-	←	4	2	3	1^-	7284.6206	-0.0145
7	2	5	1^-	←	6	1	5	1^-	7464.3509	-0.0162
4	4	0	1^-	←	3	3	0	1^-	7704.0989	0.0065
4	4	1	1^-	←	3	3	1	1^-	7704.1927	0.0064
					0^-← 0^-					
2	1	1	0^-	←	1	0	1	0^-	2348.7551	-0.0143
6	1	6	0^-	←	5	2	4	0^-	2521.0341	-0.0216
3	1	2	0^-	←	2	0	2	0^-	3245.0349	0.0356
2	2	0	0^-	←	1	1	0	0^-	3515.7611	-0.0013
4	1	3	0^-	←	3	0	3	0^-	4169.2373	0.0290
7	1	6	0^-	←	6	2	4	0^-	4299.0808	0.0015
3	2	1	0^-	←	2	1	1	0^-	4302.3199	-0.0076
3	2	2	0^-	←	2	1	2	0^-	4432.2284	0.0322
8	0	8	0^-	←	7	1	6	0^-	4889.0506	0.0232
4	2	2	0^-	←	3	1	2	0^-	5080.4709	0.0106
5	1	4	0^-	←	4	0	4	0^-	5125.9532	0.0152
4	2	3	0^-	←	3	1	3	0^-	5326.9807	0.0207
3	3	0	0^-	←	2	2	0	0^-	5619.3752	-0.0053
3	3	1	0^-	←	2	2	1	0^-	5621.9945	-0.0054
5	2	3	0^-	←	4	1	3	0^-	5859.9531	0.0150
6	1	5	0^-	←	5	0	5	0^-	6120.1601	-0.0057
5	2	4	0^-	←	4	1	4	0^-	6245.1821	0.0044

J	K_a	K_c	v	←	J'	K_a'	K_c'	v′	ν_{obs}	ν_{obs}-ν_{calc}
4	3	1	0^-	←	3	2	1	0^-	6437.8359	0.0036
4	3	2	0^-	←	3	2	2	0^-	6450.6605	-0.0053
6	2	4	0^-	←	5	1	4	0^-	6651.2984	-0.0017
7	1	6	0^-	←	6	0	6	0^-	7155.7161	-0.0274
6	2	5	0^-	←	5	1	5	0^-	7186.8476	-0.0089
5	3	2	0^-	←	4	2	2	0^-	7247.1844	0.0099
5	3	3	0^-	←	4	2	3	0^-	7284.6206	-0.0145
7	2	5	0^-	←	6	1	5	0^-	7464.4086	0.0060
4	4	0	0^-	←	3	3	0	0^-	7704.0989	0.0065
4	4	1	0^-	←	3	3	1	0^-	7704.1927	0.0064
**$1^+ \leftarrow 1^+$**										
2	1	1	1^+	←	1	0	1	1^+	2348.6899	-0.0193
6	1	6	1^+	←	5	2	4	1^+	2521.1163	-0.0063
3	1	2	1^+	←	2	0	2	1^+	3244.9077	0.0120
2	2	0	1^+	←	1	1	0	1^+	3515.7611	-0.0013
4	1	3	1^+	←	3	0	3	1^+	4169.0808	0.0185
7	1	6	1^+	←	6	2	4	1^+	4298.9844	0.0026
3	2	1	1^+	←	2	1	1	1^+	4302.3199	-0.0076
3	2	2	1^+	←	2	1	2	1^+	4432.1629	0.0106
8	0	8	1^+	←	7	1	6	1^+	4889.2154	-0.0227
4	2	2	1^+	←	3	1	2	1^+	5080.4709	0.0106
5	1	4	1^+	←	4	0	4	1^+	5125.7629	0.0092
4	2	3	1^+	←	3	1	3	1^+	5326.8717	0.0147
3	3	0	1^+	←	2	2	0	1^+	5619.3752	-0.0053
3	3	1	1^+	←	2	2	1	1^+	5621.9945	-0.0054
5	2	3	1^+	←	4	1	3	1^+	5859.9531	0.0150
6	1	5	1^+	←	5	0	5	1^+	6119.9534	0.0004
5	2	4	1^+	←	4	1	4	1^+	6245.0252	-0.0087
4	3	1	1^+	←	3	2	1	1^+	6437.8359	0.0036
4	3	2	1^+	←	3	2	2	1^+	6450.6605	-0.0053
6	2	4	1^+	←	5	1	4	1^+	6651.2984	-0.0017
7	1	6	1^+	←	6	0	6	1^+	7155.5040	-0.0156
6	2	5	1^+	←	5	1	5	1^+	7186.6632	-0.0149
5	3	2	1^+	←	4	2	2	1^+	7247.1844	0.0099
5	3	3	1^+	←	4	2	3	1^+	7284.6206	-0.0145
7	2	5	1^+	←	6	1	5	1^+	7464.4086	0.0060
4	4	0	1^+	←	3	3	0	1^+	7704.0989	0.0065
4	4	1	1^+	←	3	3	1	1^+	7704.1927	0.0064
**^cQ-branch**										
**$0^+ \leftarrow 0^+$**										
7	3	5	0^+	←	7	2	5	0^+	2927.2638	0.0152
5	3	3	0^+	←	5	2	3	0^+	3080.8910	-0.0016
9	4	6	0^+	←	9	3	6	0^+	4307.7212	0.0009
8	4	5	0^+	←	8	3	5	0^+	4349.6042	-0.0033
7	4	4	0^+	←	7	3	4	0^+	4375.7497	0.0056
6	4	3	0^+	←	6	3	3	0^+	4391.2035	-0.0025
8	4	4	0^+	←	8	3	6	0^+	4394.0782	-0.0047
9	4	5	0^+	←	9	3	7	0^+	4395.5751	-0.0003
7	4	3	0^+	←	7	3	5	0^+	4396.0887	-0.0099
6	4	2	0^+	←	6	3	4	0^+	4399.3663	-0.0008
5	4	2	0^+	←	5	3	2	0^+	4399.7384	0.0021
5	4	1	0^+	←	5	3	3	0^+	4402.4668	0.0109
8	5	3	0^+	←	8	4	5	0^+	5651.9883	0.0031
7	5	3	0^+	←	7	4	3	0^+	5657.0295	0.0198
7	5	2	0^+	←	7	4	4	0^+	5657.4825	-0.0010
**$1^- \leftarrow 1^-$**										
7	3	5	1^-	←	7	2	5	1^-	2927.2638	0.0152
5	3	3	1^-	←	5	2	3	1^-	3080.9560	0.0019
9	4	6	1^-	←	9	3	6	1^-	4307.7212	0.0009

J	K_a	K_c	v	←	J'	K_a'	K_c'	v′	ν_{obs}	$\nu_{obs}-\nu_{calc}$
8	4	5	1^-	←	8	3	5	1^-	4349.6042	-0.0033
7	4	4	1^-	←	7	3	4	1^-	4375.7780	0.0043
6	4	3	1^-	←	6	3	3	1^-	4391.2035	-0.0025
8	4	4	1^-	←	8	3	6	1^-	4394.0782	-0.0047
9	4	5	1^-	←	9	3	7	1^-	4395.5751	-0.0003
7	4	3	1^-	←	7	3	5	1^-	4396.0887	-0.0099
6	4	2	1^-	←	6	3	4	1^-	4399.3663	-0.0008
5	4	2	1^-	←	5	3	2	1^-	4399.8050	0.0106
5	4	1	1^-	←	5	3	3	1^-	4402.5126	-0.0012
8	5	3	1^-	←	8	4	5	1^-	5651.9883	0.0031
7	5	3	1^-	←	7	4	3	1^-	5657.0295	0.0198
7	5	2	1^-	←	7	4	4	1^-	5657.4825	-0.0010
					**$0^- \leftarrow 0^-$**					
7	3	5	0^-	←	7	2	5	0^-	2927.2638	0.0152
5	3	3	0^-	←	5	2	3	0^-	3080.8910	-0.0016
9	4	6	0^-	←	9	3	6	0^-	4307.7212	-0.0138
8	4	5	0^-	←	8	3	5	0^-	4349.6042	-0.0033
7	4	4	0^-	←	7	3	4	0^-	4375.7497	0.0056
6	4	3	0^-	←	6	3	3	0^-	4391.2035	-0.0025
8	4	4	0^-	←	8	3	6	0^-	4394.0782	-0.0047
9	4	5	0^-	←	9	3	7	0^-	4395.5751	-0.0003
7	4	3	0^-	←	7	3	5	0^-	4396.1221	0.0010
6	4	2	0^-	←	6	3	4	0^-	4399.3663	-0.0008
5	4	2	0^-	←	5	3	2	0^-	4399.7384	-0.0195
5	4	1	0^-	←	5	3	3	0^-	4402.4668	-0.0115
8	5	3	0^-	←	8	4	5	0^-	5651.9883	0.0031
7	5	3	0^-	←	7	4	3	0^-	5657.0295	0.0198
7	5	2	0^-	←	7	4	4	0^-	5657.4825	-0.0010
					**$1^+ \leftarrow 1^+$**					
7	3	5	1^+	←	7	2	5	1^+	2927.2638	0.0152
5	3	3	1^+	←	5	2	3	1^+	3080.9560	0.0019
9	4	6	1^+	←	9	3	6	1^+	4307.7212	0.0009
8	4	5	1^+	←	8	3	5	1^+	4349.6042	-0.0033
7	4	4	1^+	←	7	3	4	1^+	4375.7780	0.0043
6	4	3	1^+	←	6	3	3	1^+	4391.2035	-0.0025
8	4	4	1^+	←	8	3	6	1^+	4394.0782	-0.0047
9	4	5	1^+	←	9	3	7	1^+	4395.5751	-0.0003
7	4	3	1^+	←	7	3	5	1^+	4396.1221	0.0010
6	4	2	1^+	←	6	3	4	1^+	4399.3663	-0.0008
5	4	2	1^+	←	5	3	2	1^+	4399.8050	-0.0106
5	4	1	1^+	←	5	3	3	1^+	4402.5126	-0.0246
8	5	3	1^+	←	8	4	5	1^+	5651.9883	0.0031
7	5	3	1^+	←	7	4	3	1^+	5657.0295	0.0198
7	5	2	1^+	←	7	4	4	1^+	5657.4825	-0.0010

References

[1] D. Schmitz, V. Alvin Shubert, T. Betz, and M. Schnell, “Multi-resonance effects within a single chirp in broadband rotational spectroscopy: The rapid adiabatic passage regime for benzonitrile,” *Journal of Molecular Spectroscopy*, vol. 280, pp. 77–84, Oct. 2012, doi: 10.1016/j.jms.2012.08.001.

[2] C. Pérez, A. Krin, A. L. Steber, J. C. López, Z. Kisiel, and M. Schnell, “Wetting Camphor: Multi-Isotopic Substitution Identifies the Complementary Roles of Hydrogen Bonding and Dispersive Forces,” *J. Phys. Chem. Lett.*, vol. 7, no. 1, pp. 154–160, Jan. 2016, doi: 10.1021/acs.jpclett.5b02541.

[3] D. Loru *et al.*, “How does the composition of a PAH influence its microsolvation? A rotational spectroscopy study of the phenanthrene–water and phenanthridine–water clusters,” *Phys. Chem. Chem. Phys.*, vol. 23, no. 16, pp. 9721–9732, Apr. 2021, doi: 10.1039/D1CP00898F.

[4] R. Meyer, “Flexible models for intramolecular motion, a versatile treatment and its application to glyoxal,” *Journal of Molecular Spectroscopy*, vol. 76, no. 1, pp. 266–300, Jun. 1979, doi: 10.1016/0022-2852(79)90230-3.
